# Supplementary material for: Ranolazine rescues the heart failure phenotype of PLN-deficient human pluripotent stem cell-derived cardiomyocytes
Source: Stem Cell Reports. 2022 Mar 24;17(4):804–19. doi: 10.1016/j.stemcr.2022.02.016 (PMC9023809; doi:10.1016/j.stemcr.2022.02.016)
Supplement: Document S1. Figures S1–S6 and Tables S1 and S2 [file mmc1.pdf]

Stem Cell Reports, Volume *17*

Supplemental Information

Ranolazine rescues the heart failure phenotype of *PLN*-deficient human pluripotent stem cell-derived cardiomyocytes

Youxu Jiang, Xiaowei Li, Tianwei Guo, Wen-Jing Lu, Shuhong Ma, Yun Chang, Yuanxiu Song, Siyao Zhang, Rui Bai, Hongyue Wang, Man Qi, Hongfeng Jiang, Hongjia Zhang, and Feng Lan

Supplementary Figure 1:Characterization of *PLN* KO hiPSCs and hiPSC-CM differentiation efficiency.Method details on the transcript GCaMP-expression of *PLN* KO hiPSCs

A 46 X, X normal karyotype of *PLN* KO hiPSCs

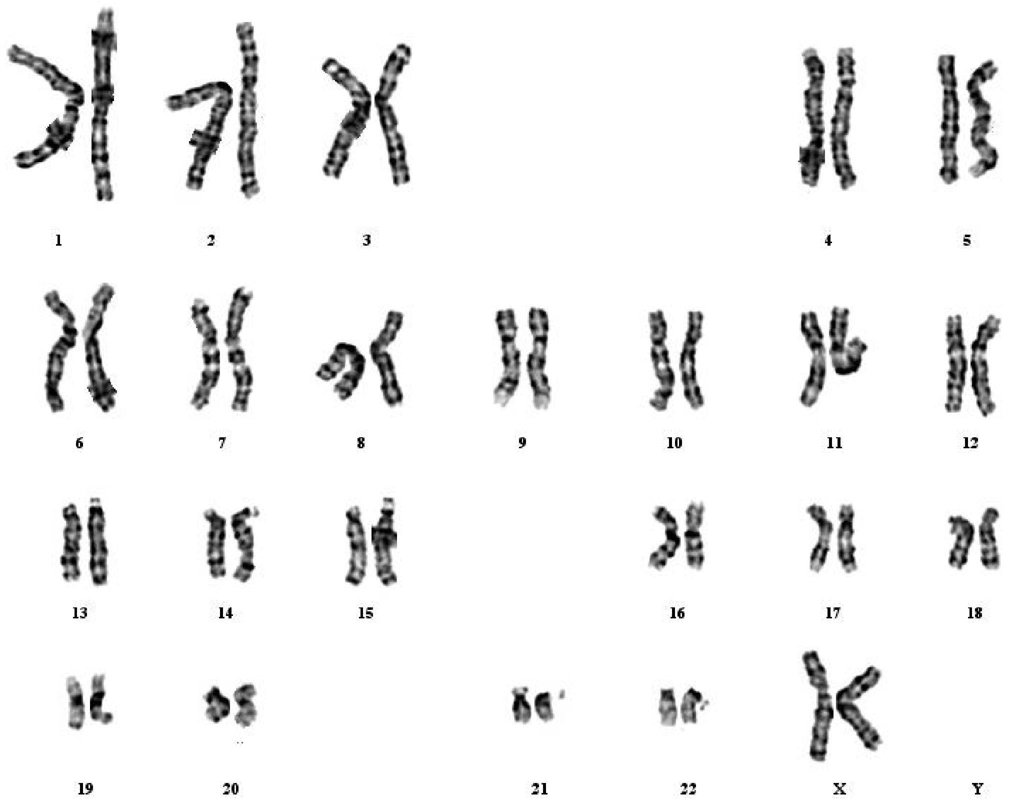

B Teratomas contain cell lineages from three germ layers (ectoderm, mesoderm and endoderm). Scale bars, 100μm.

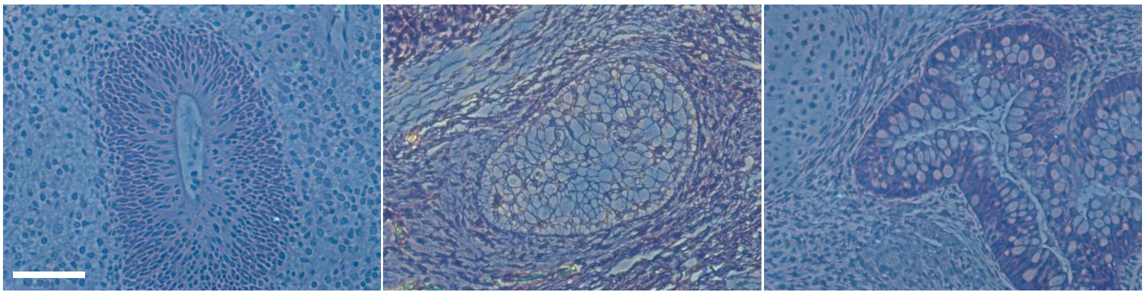

ectoderm **mesoderm** **endoderm**

C Protocol for cardiac differentiation using small molecule-based methods

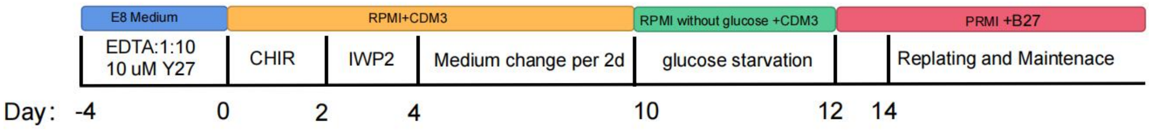

D:Schematic demonstrating the GCaMP-expression cassette that was integrated into AAVS1 of WT and *PLN* KO hiPSCs via nickase CRISPR/Cas9 editing.

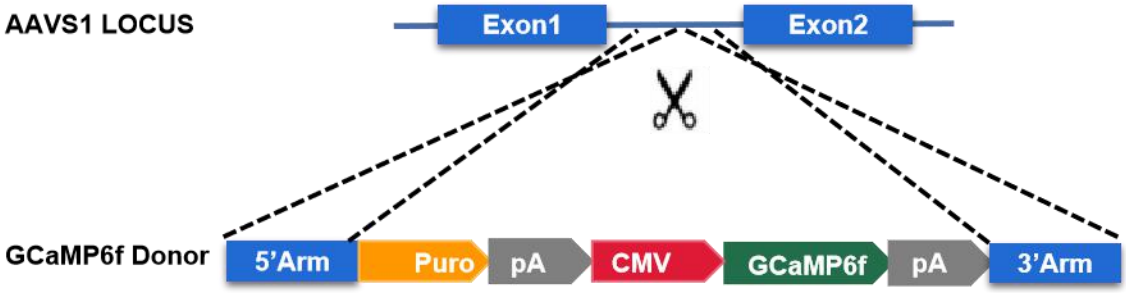

E:Space-averaged calcium transients showing parameters measured for analysis of calcium handling

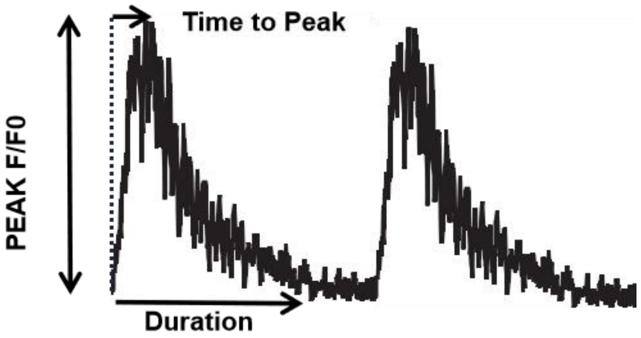

F:Sequence chromatograms demonstrate a Heterozygote *PLN* gene knockout line which one allely deleted 7 nucleotide(ACTTGCT).

	S	N	T	S	L	A	Q
WT	TCC	AAT	ACC	TCA	CTC	GCT	CAG
	S	N	T	S	S	Y	K
Heterozygote	TCC	AAT	ACC	TCC	AGC	TAT	AAG

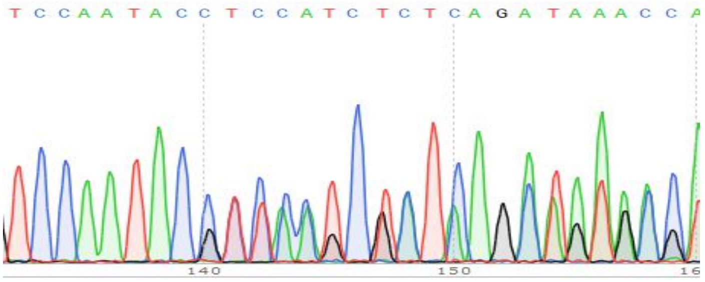

G:Quantification of *PLN* normalized by GAPDH in WT and *PLN* heterozygote hiPSC-CMs

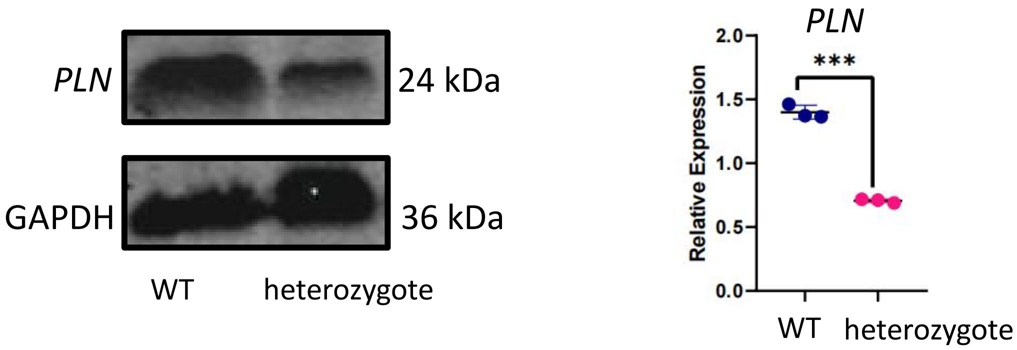

Supplementary Figure2:（A-D）the results of contractility in the presence of WT ,

***PLN*-KO,WT+ISO(500nM) and *PLN*-KO+ISO(E-H)the results of calcium transient in the**

presence of WT,*PLN*-KO,WT+ISO(500nM) and *PLN*-KO+ISO

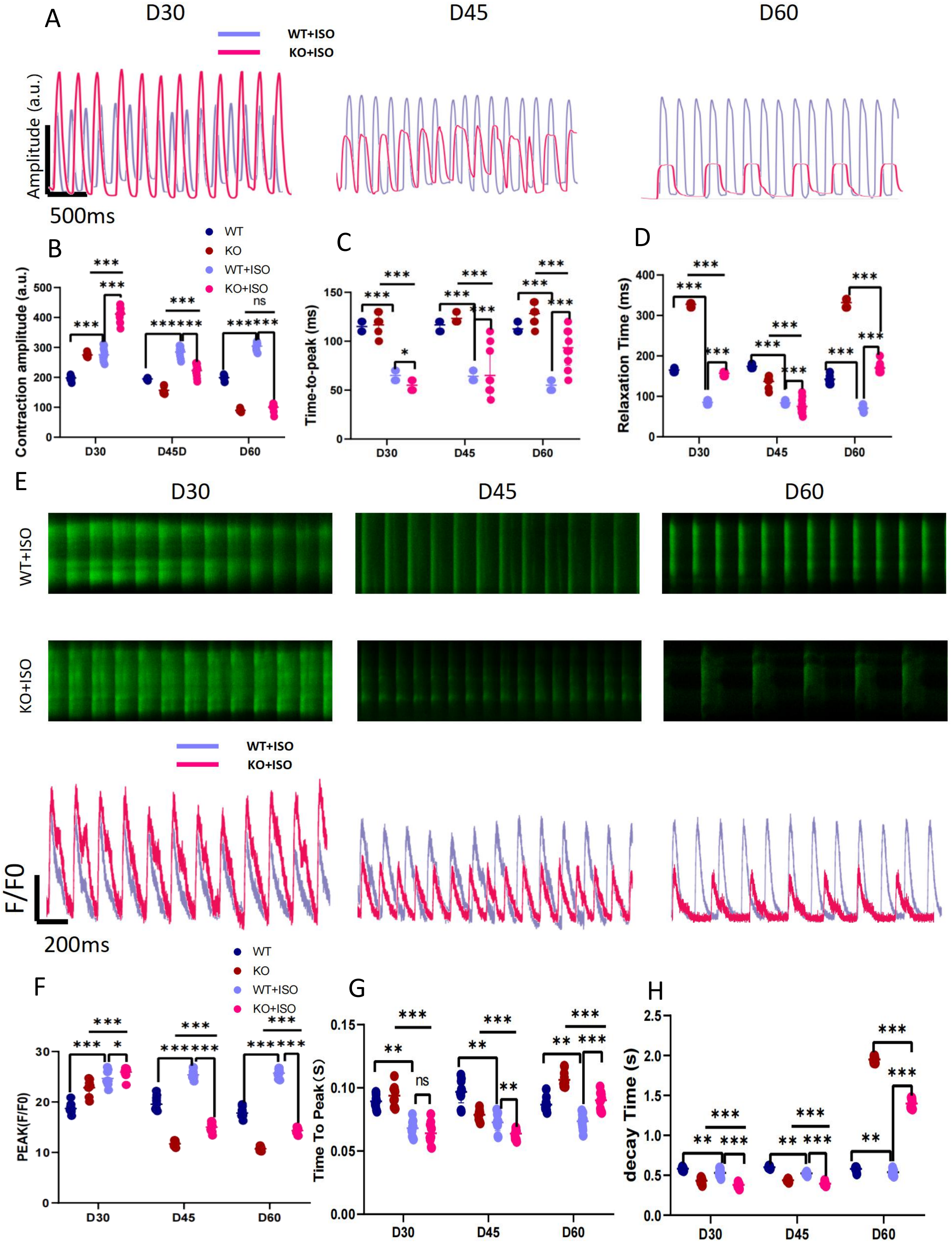

Supplementary Figure 3:(A) The Relative protein expression levels of cl-PARP /p-AKT/AKT/BCL-2/ cl-caspapse3 /bax at day 60;(B)The Relative protein expression levels of cl-PARP /p-AKT/AKT/BCL-2/ cl-caspapse3 /BAX with ranolazine at day 60（C）A Gene Set Enrichment Analysis (GSEA) revealed that pathways

A:

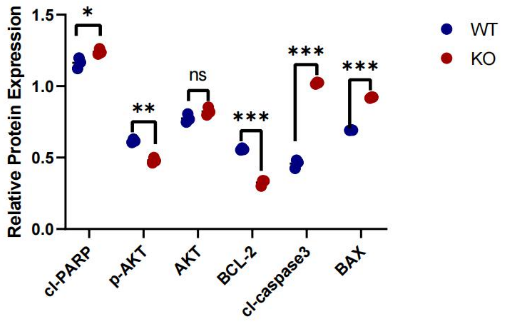

B:

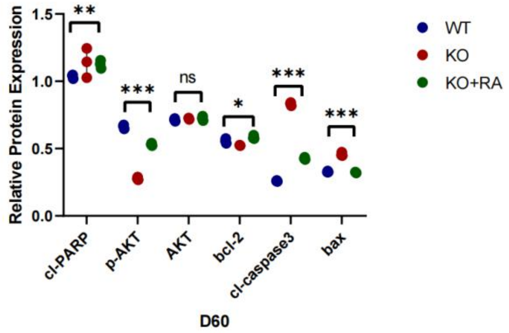

C:

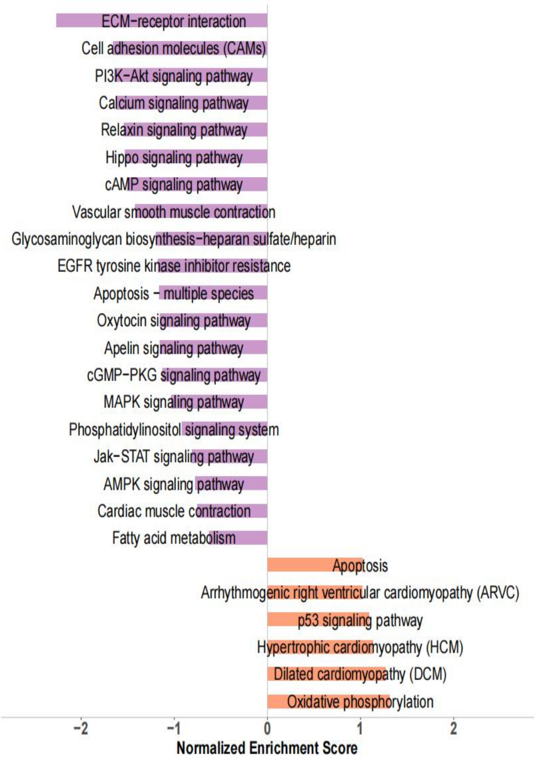

Supplementary Figure 4. Ranolazine recovered calcium transport disorders and myocardial contractility in *PLN*-KO hiPSC-CMs at day 30d and day 45.

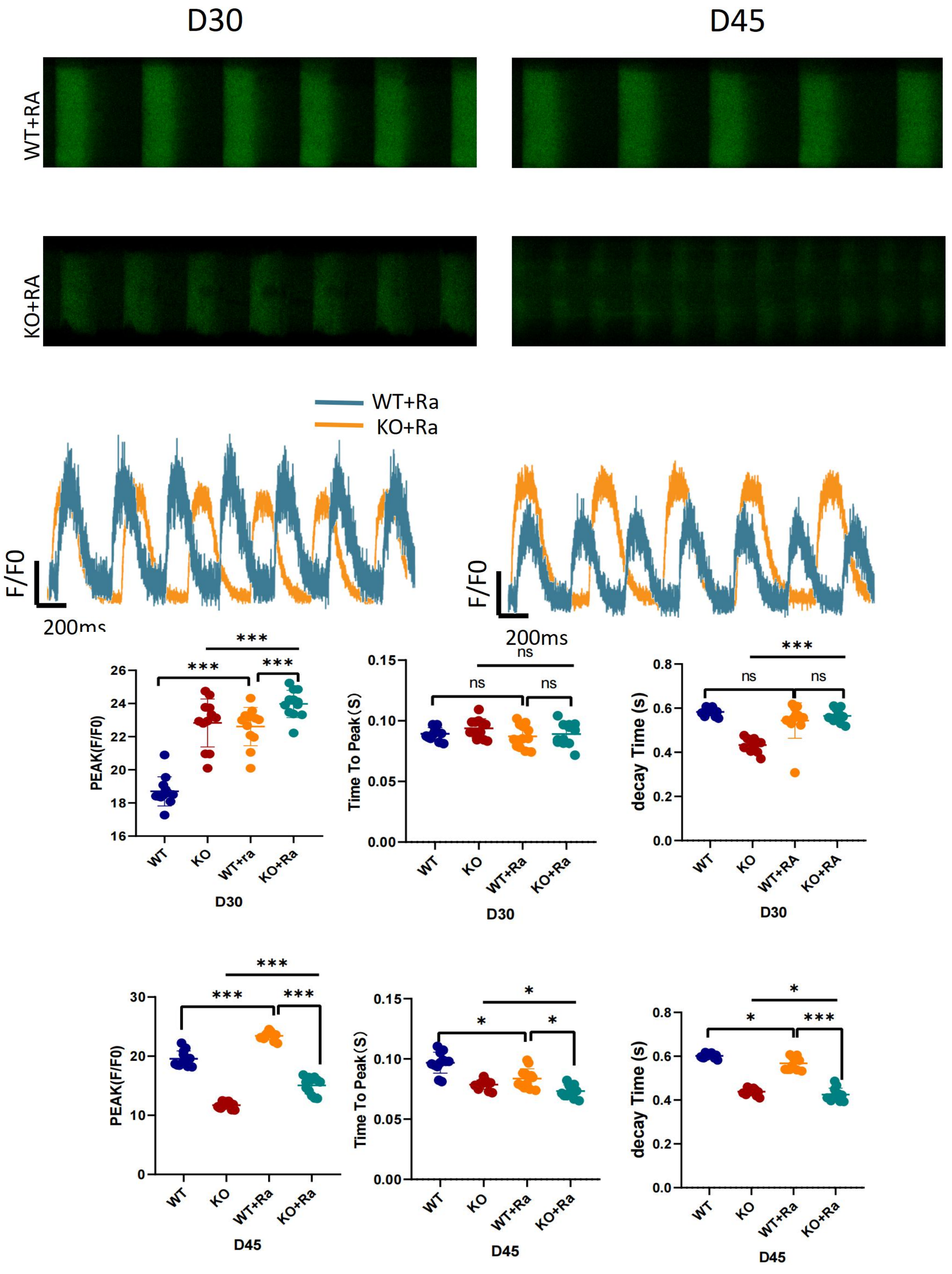

Supplementary Figure 5:Ranolazine recovered mitochondrial dysfunction and cell activates oxidative stress in *PLN*-KO hiPSC-CMs (a,b)Assessment of oxygen consumption rate (OCR) was performed with the application of compounds to assess ATP production (Oligomycin), maximum (Max) respiration (FCCP, carbonyl cyanide-4 [trifluoromethoxy] phenylhydrazone) and halted respiration (antimycin A and rotenone) for WT,*PLN* KO,WT+ranolazine and *PLN* KO+ranolazine hiPSC-CMs at day 30 and day 45(n=12)（C）Basal respiration of WT,*PLN* KO,WT+ranolazine and *PLN* KO+ranolazine hiPSC-CMs at day 30,45,60 (D) maximum respiration of WT,*PLN* KO,WT+ranolazine and *PLN* KO+ranolazine hiPSC-CMs at day 30,45,60(J) the ATP level in WT and *PLN* KO hiPSC-CMs at day 30,45,60.（E-J）The relative expression of glucose metabolism and fatty acid metabolism with ranolazine at day 60.

A:

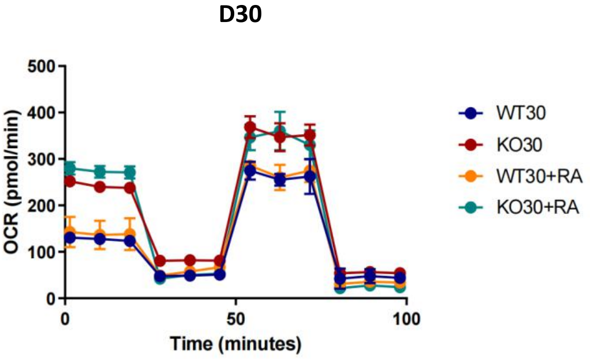

B:

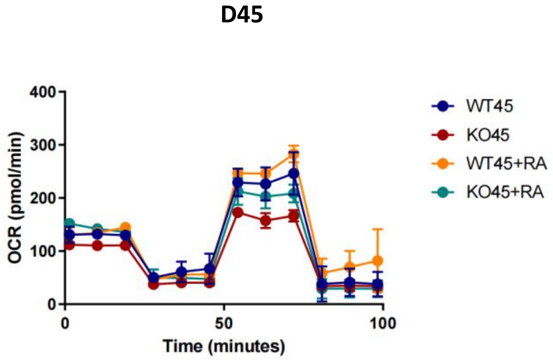

C

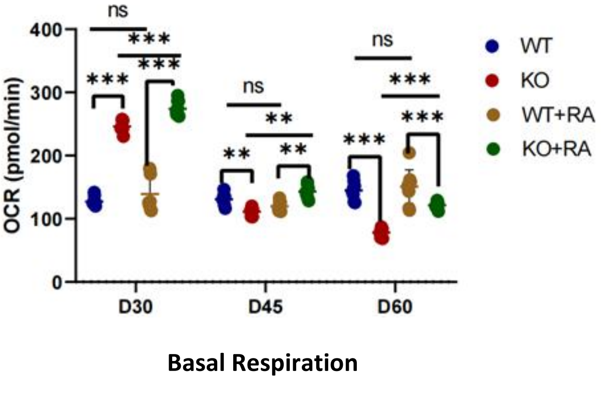

D:

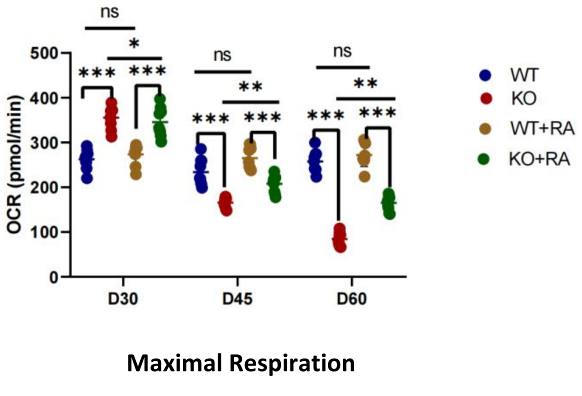

E

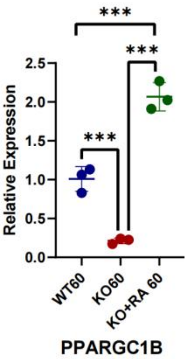

F

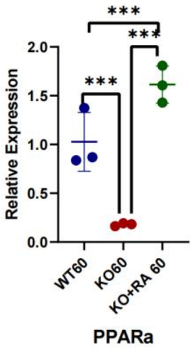

G

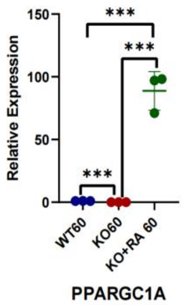

H

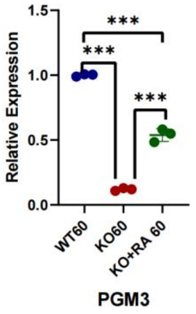
1.5
1.0
0.5
0.0
Relative Expression
***
***
***
WT60
KO60
KO+RA 60
PGM3

I

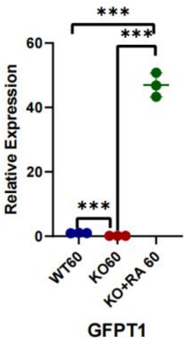
60
40
20
0
Relative Expression
***
***
***
WT60
KO60
KO+RA 60
GFPT1

J

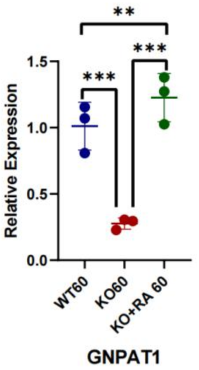
**
1.5
1.0
0.5
0.0
Relative Expression
***
***
WT60
KO60
KO+RA 60
GNPAT1

Supplementary Figure 6:(A)Representative line-scan images from WT , *PLN* heterozygotes (+/-) hiPSC-CMs after 1 week of 1 μ M ISO treatment beginning at day 53 of cardiac differentiation. (B-D)Quantifification of peak, time to peak, and calcium decay time in WT-GCaMP+ISO and *PLN* heterozygotes（+/-）-GCaMP+ISO hiPSC-CMs at day 60 (n = 12 cells per group);（E）Representative line-scan images from WT+ISO , *PLN* heterozygotes (+/-) +ISO hiPSC-CMs after 5 day of 1 μ M ranolazine treatment.(F-H)Quantifification of peak, time to peak, and calcium decay time in WT,*PLN* heterozygotes+ISO,WT-GCaMP+ISO+Ra and *PLN* heterozygotes（+/-）-GCaMP+ISO+Ra hiPSC-CMs at day 60 (n = 12 cells per group);

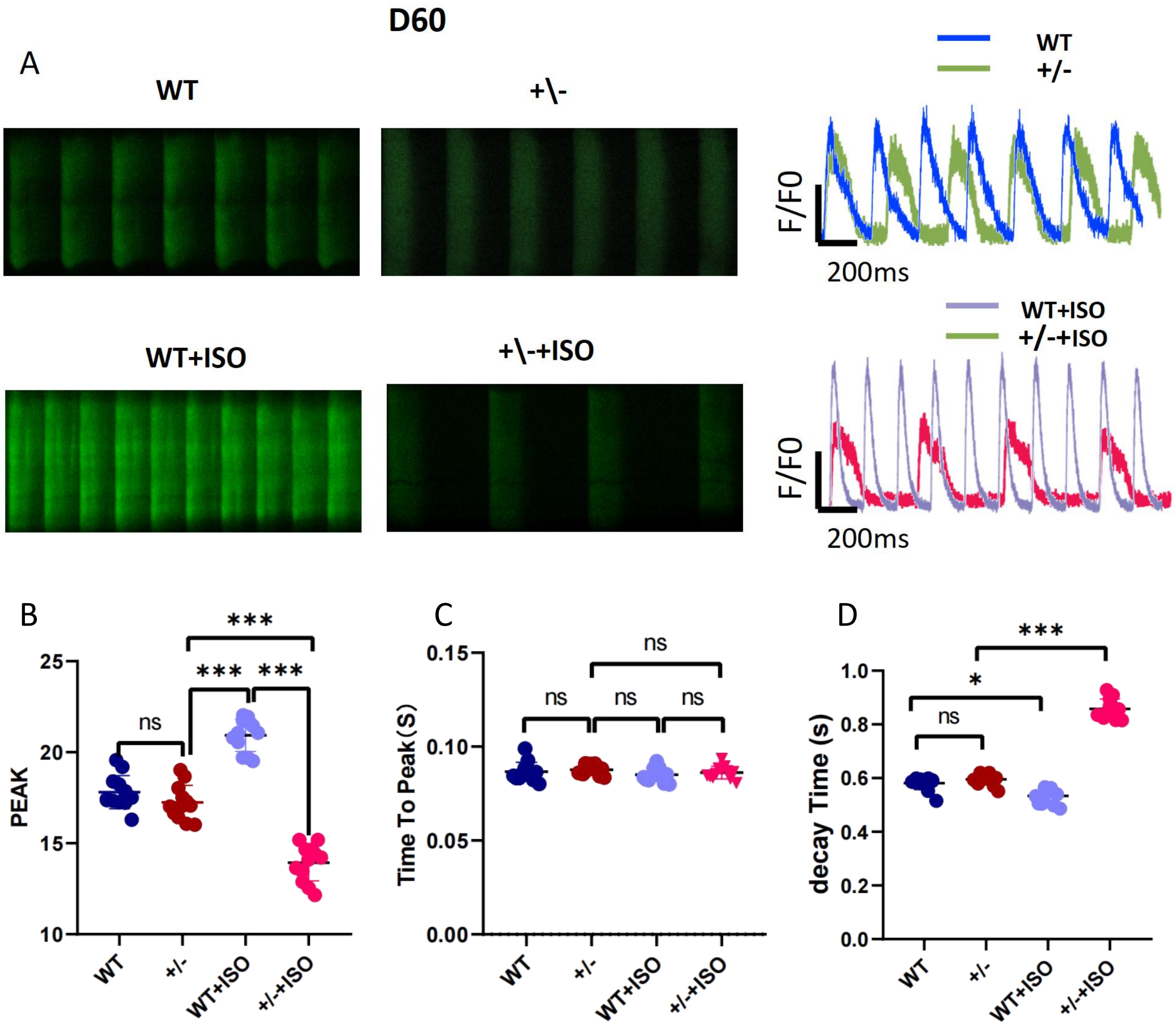

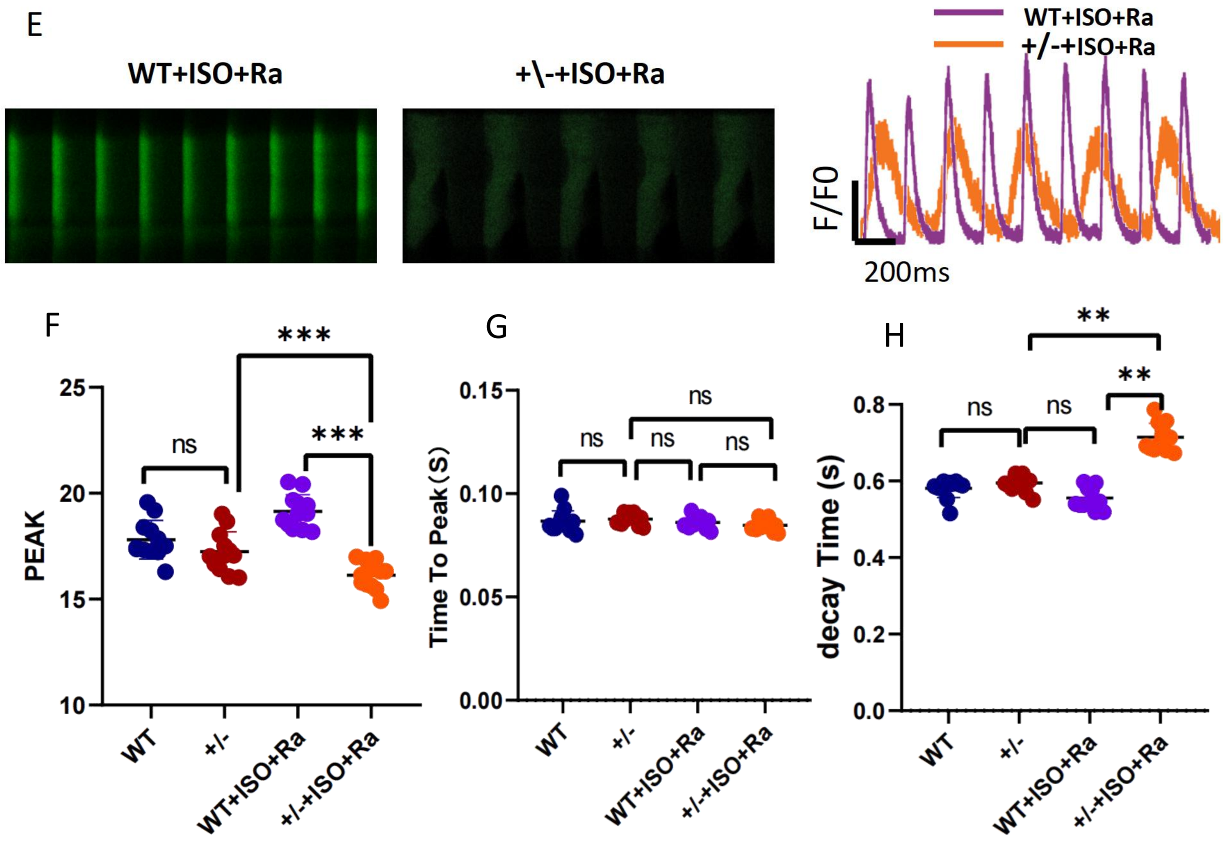
E
WT+ISO+Ra
+\-+ISO+Ra
WT+ISO+Ra
+/-+ISO+Ra
F/F0
200ms
F
PEAK
25
20
15
10
ns
***
***
WT
+/-
WT+ISO+Ra
+/-+ISO+Ra
G
Time To Peak (S)
0.15
0.10
0.05
0.00
ns
ns
ns
ns
WT
+/-
WT+ISO+Ra
+/-+ISO+Ra
H
decay Time (s)
0.8
0.6
0.4
0.2
0.0
**
**
ns
ns
WT
+/-
WT+ISO+Ra
+/-+ISO+Ra

Table1：Primer sequences used for q-PCR

Gene	Forward 5'-3'	Reverse 5'-3'
ND1	ATGGCCAACCTCCTACTCCTCATT	TTATGGCGTCAGCGAAGGGTTGTA
ND2	CCATCTTTGCAGGCACACTCATCA	ATTATGGATGCGGTTGCTTGCGTG
ACTB	CATGTACGTTGCTATCCAGGC	CTCCTTAATGTCACGCACGAT
MYH6	TCTCCGACAACGCCTATCAGTAC	GTCACCTATGGCTGCAATGCT
MYH7	GGCAAGACAGTGACCGTGAAG	CGTAGCGATCCTTGAGGTTGTA
ANP	ACAATGCCGTGTCCAACGCAGA	CTTCATTCGGCTCACTGAGCAC
BNP	TCTGGCTGCTTTGGGAGGAAGA	CCTTGTGGAATCAGAAGCAGGTG
TNNT2	TTCACCAAAGATCTGCTCCTCGCT	TTATTACTGGTGTGGAGTGGGTGTGG
ACTA1	AGGTCATCACCATCGGCAACGA	GCTGTTGTAGGTGGTCTCGTGA
RYR2	AGAACTTACACACGCGACCTG	CATCTCTAACCGGACCATACTGC
PLN	AGCACGTCAAAAGCTACAGAATCT	CTGATGTGGCAAGCTGCAGATC
GAPDH	*GGAGCGAGATCCCTCCAAAAT*	*GGCTGTTGTCATACTTCTCATGG*
PPARA	*TCGGCGAGGATAGTTCTGGAAG*	*GACCACAGGATAAGTCACCGAG*
PPARGC1A	*CCAAAGGATGCGCTCTCGTTCA*	*CGGTGTCTGTAGTGGCTTGACT*
PPARGC1B	*TGAGCAGACCTTGACAGTGGAG*	*GACTATGCTTGATGTCTGGTTTGA*
GNPAT1	*CCAACACATCCTGGAGAAGGCT*	*GGCTGACAACTCCAGTCTCTGT*
PGM3	*GGAGGCAATCAATGACCTGGTG*	*CCAAGCTCACTTCATGTGCAAGG*
GFPT1	*CCCTCTGTTGATTGGTGTACGG*	*GGAAAAGGCAGGTTGTGCTGTC*

Table2：Primary and Secondary Antibodies

Type	Antibody	Application	Dilution	Species	Manufacturer And Catalog Number
Primary	Anti-OCT4	Immunofluorescence	1:100	Rabbit Polyclonal	Santa Cruz sc-9081
	Anti-TRA-1-8 1	Immunofluorescence	1:100	Mouse Monoclonal	Santa Cruz sc-21706
	Anti-BAX	Western blot	1:1000	Rabbit Polyclonal	Abcam Ab182733
	Caspase3/P17/19 Polyclonal antibody	Western blot	1:1000	Rabbit Monoclonal	proteinTech 19677-1-AP
	PARP1 Polyclonal antibody	Western blot	1:1000	Rabbit Monoclonal	proteinTech 13371-1-AP
	Anti-*PLN*	Immunofluorescence	1:100	Mouse Monoclonal	Santa Cruz sc-393990
		Western blot	1:1000		
	Anti-cTnT	Immunofluorescence	1:100	Mouse Monoclonal	Abcam Ab8295
		Western blot	1:1000		
		Flow cytometry	1:200		
	AKT Polyclonal antibody	Western blot	1:1000	Rabbit Monoclonal	proteinTech 10176-2-AP
	Phospho-AKT(Ser473) Monoclonal antibody	Western blot	1:1000	Mouse Monoclonal	proteinTech 66444-1-Ig
	BCL-2 Polyclonal antibody	Western blot	1:1000	Rabbit Polyclonal	proteinTech 12789-1-AP
	Anti-GAPDH	Western blot	1:1000	Mouse Monoclonal	Santa Cruz sc-365062
Secondary	Goat anti-Mouse IgG Alexa Fluor 594	Immunofluorescence	1:200	Goat anti-Mouse IgG	Invitrogen A21145
	Goat anti-Rabbit IgG Alexa Fluor 488	Immunofluorescence	1:200	Goat anti-Rabbit IgG	Invitrogen A32731
	Chicken anti-Rabbit IgG	Immunofluorescence	1:200	Chicken anti-Rabbit IgG	Invitrogen A21442

Secondary	Alexa Fluor 594				
	Chicken anti-Mouse IgG Alexa Fluor 488	Immunofluorescence	1:200	Chicken anti-Mouse IgG	Invitrogen A21200
	Goat anti-Rabbit IgG (H + L) IRDye 800CW	Western blot	1:20000	Goat anti-Rabbit IgG	LI-COR 926-32211
	Goat anti-Mouse IgG (H + L) IRDye 800CW	Western blot	1:20000	Goat anti-Mouse IgG	LI-COR 926-32210

华大基因
BGI

Brief Report

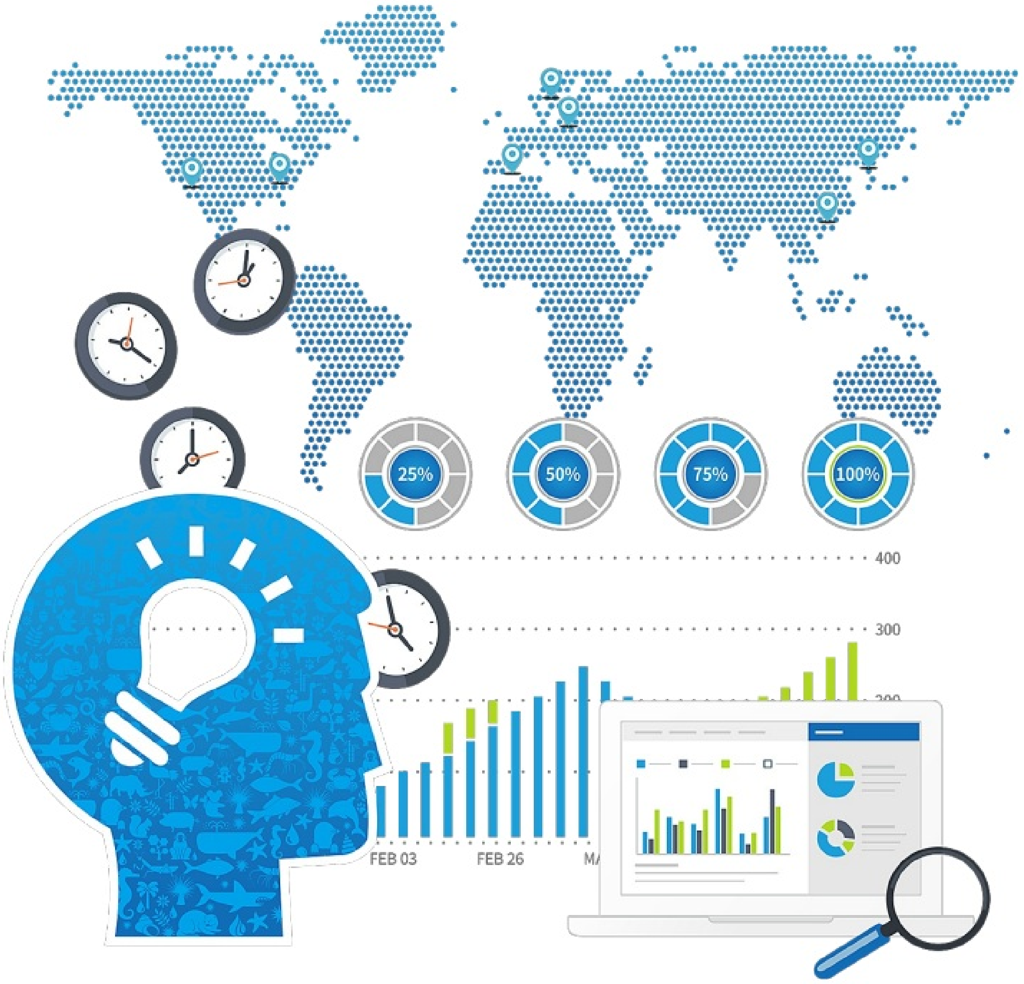

Catalogue

Catalogue 1
1 Project overview 2
1.1 Abstract 2
1.2 Analysis Plan 2
1.2.1 Setting of sample distribution 2
1.2.2 Differential grouping set 2
1.2.3 Time series analysis scheme 2
1.3 Sample correlation 3
1.3.1 Sample correlation 3
1.4 Expression quantity distribution 3
1.4.1 Boxplot 3
1.4.2 Gene expression density map 4
2 Reference Information 4
2.1 Reference species 4
2.2 RNA classification 4
2.3 Gene length 4
2.4 Number of exons 5
3 Reads filtering 6
3.1 Quality statistics of filtered reads 6
3.1.1 Reads filtering 6
3.2 Statistics of raw data 7
4 Reads Align 7
4.1 Align to the reference genome 7
4.2 Align to the reference gene 8
5 Significant DEGs 9

1 Project overview

Project number: F20FTSCCWLJ6851_HOwksE

1.1 Abstract

In this project, we sequence 12 samples used BGISEQ platform, averagely generating about 6.66G Gb bases per sample. The average mapping ratio with reference genome is 87.77%, the average mapping ratio with gene is 68.68%; 18191 genes were identified.

1.2 Analysis Plan

1.2.1 Setting of sample distribution

Biological experiments generally have biological replicates. Biological replicates generally occurs in biological experiment, and the multiple samples of biological replicates shall be classified into one group. The following table is the grouping of samples used in analysis:

ID	Group Name	Sample Name
1	WT45D	WT_45D_2,WT_45D_1,WT_45D_3
2	KO60D	KO_60D_1,KO_60D_2,KO_60D_3
3	WT60D	WT_60D_2,WT_60D_1,WT_60D_3
4	KO45D	KO_45D_2,KO_45D_1,KO_45D_3

1.2.2 Differential grouping set

One of the important analytic points of transcriptome sequencing is the comparison of the differences in gene expression. The control and treatment settings in pairwise comparison are as follows:

ID	Control Group	Treat Group
1	WT45D	WT60D
2	WT60D	KO60D
3	WT45D	KO45D
4	KO45D	KO60D

1.2.3 Time series analysis scheme

The correspondence between the name of the time series analysis plan and the sample:

No Result

1.3 Sample correlation

In order to reflect the correlation of gene expression between samples, the Pearson correlation coefficients of all gene expressions between each two samples were calculated, and these coefficients were reflected in the form of a heatmap. The correlation coefficients can reflect the similar situation of the overall gene expression between each sample. The higher the correlation coefficient is, the more similar the gene expression level is.

1.3.1 Sample correlation

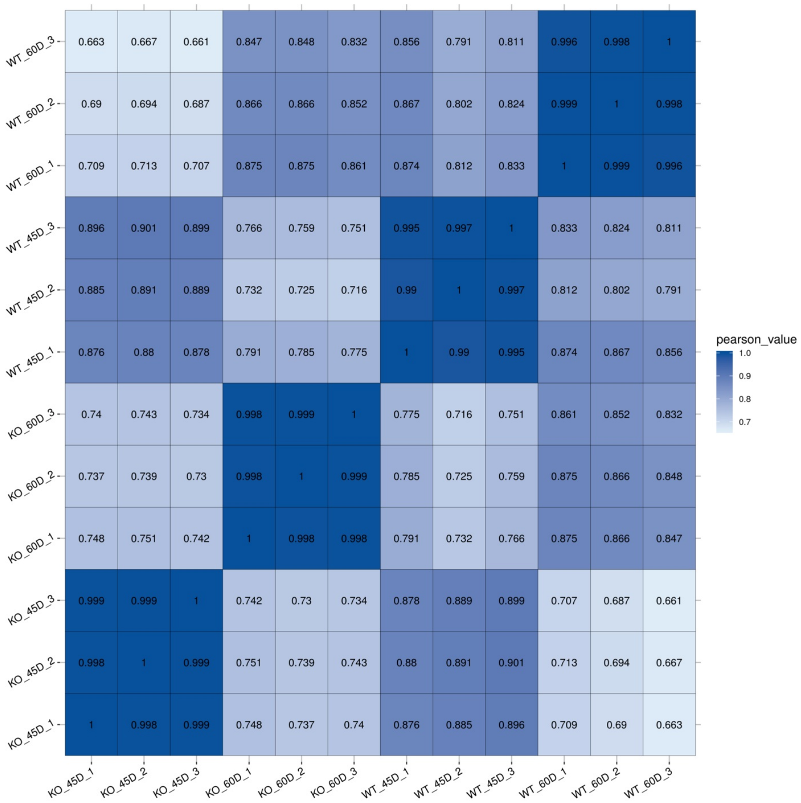

1.4 Expression quantity distribution

1.4.1 Boxplot

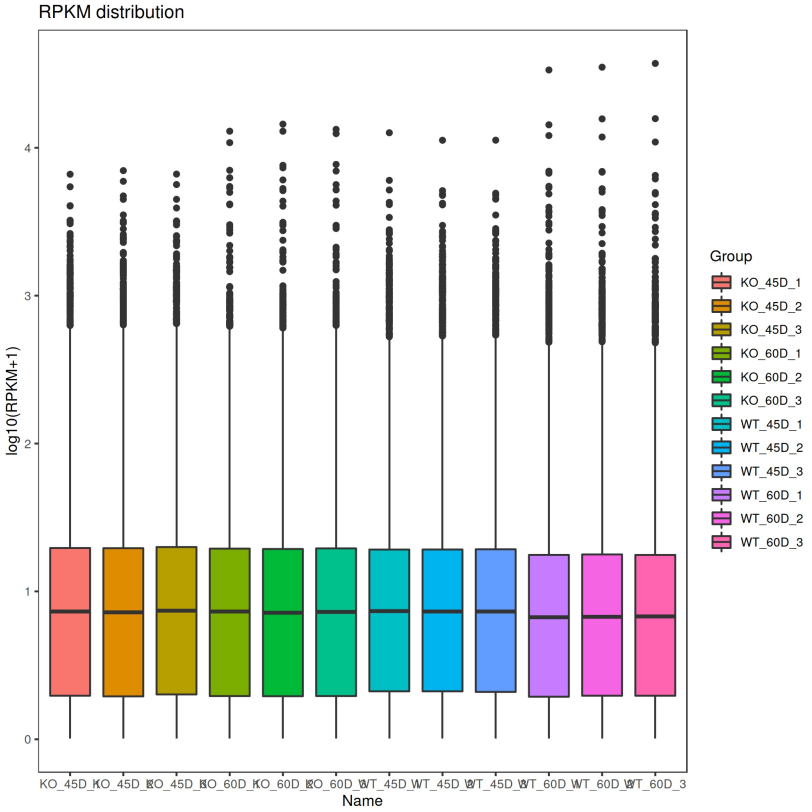

1.4.2 Gene expression density map

To show the gene amount under different FPKM value, we calculate the gene amount under three different FPKM ranges(FPKM <= 1、FPKM 1~10、FPKM >= 10).

No Result

2 Reference Information

2.1 Reference species

Species: Homo_sapiens

Source: NCBI

Reference Genome Version: GCF_000001405.38_GRCh38.p12

2.2 RNA classification

mRNA Num	lncRNA Num	miRNA Num
230487	177880	2656

2.3 Gene length

RNA Length	All	mRNA	mRNA	lncRNA	lncRNA

Item	RNA	Known	Novel	Known	Novel
0-500	53781	461	3846	34654	14820
500-1000	67098	5284	8233	42293	11288
1000-1500	40333	9629	8374	16902	5428
1500-2000	35301	13624	7816	11384	2477
2000-2500	31335	14418	7122	8484	1311
2500-3000	26160	13114	6628	5615	803
3000-3500	21427	10949	5945	4042	491
3500-4000	18783	9958	5443	3071	311
4000-4500	15620	8298	4805	2282	235
4500-5000	12960	6596	4440	1749	175
5000-5500	10917	5433	3933	1428	123
5500-6000	9237	4539	3551	1056	91
6000-6500	8133	4008	3123	921	81
6500-7000	6692	3063	2770	783	76
7000-7500	5673	2476	2516	616	65
7500-8000	5039	2260	2218	523	38
8000-8500	4427	1870	2030	483	44
8500+	35451	7744	23970	3457	280

2.4 Number of exons

Exon Num Item	All RNA	mRNA Known	mRNA Novel	lncRNA Known	lncRNA Novel
1	61395	1513	7643	25720	26519
2	60469	3231	10986	39494	6758
3	46715	5136	7913	31409	2257
4	32360	6819	7042	17448	1051

5	24276	7467	6537	9679	593
6	19218	7529	5933	5434	322
7	15897	7211	5397	3086	203
8	13975	7014	4955	1877	129
9	12642	6785	4491	1273	93
10+	121420	71019	45866	4323	212

3 Reads filtering

Sequencing Platform: BGISEQ

Sequencing length: PE150

The raw data for sequencing contains reads of low quality, connector contamination, and excessively high levels of unknown base N. These reads need to be removed before data analysis to ensure the reliability of the results.

3.1 Quality statistics of filtered reads

3.1.1 Reads filtering

Sample Name	Total Raw Reads	Total Clean Reads	Total Clean Bases	Clean Reads Q20	Clean Reads Q30	Clean Reads Ratio
KO_45D_1	45.57	44.61	6.69	94.63	88.17	97.88
KO_45D_2	45.57	44.47	6.67	94.44	87.96	97.57
KO_45D_3	44.13	42.53	6.38	94.53	88.12	96.37
KO_60D_1	45.57	44.53	6.68	94.42	87.89	97.72
KO_60D_2	49.08	44.62	6.69	94.90	88.93	90.91
KO_60D_3	47.33	45.43	6.81	94.63	88.38	95.99
WT_45D_1	45.57	44.66	6.70	94.62	88.13	97.99
WT_45D_2	45.57	43.90	6.58	95.03	88.98	96.32
WT_45D_3	47.33	45.42	6.81	94.70	88.51	95.96
WT_60D_1	45.57	44.48	6.67	94.52	88.07	97.61

WT_60D_2	45.57	44.33	6.65	94.68	88.41	97.28
WT_60D_3	45.57	44.16	6.62	94.45	87.97	96.91

3.2 Statistics of raw data

Sample Name	N Read Num	Adapter Read Num	Low Quality Read Num	Clean Read Num
WT_45D_2	161510	1513732	102	43898548
KO_45D_3	197292	1403626	136	42530452
WT_60D_2	198608	1041796	116	44333372
WT_45D_3	204096	1705868	104	45416666
WT_45D_1	149598	767990	128	44656176
KO_60D_1	193790	847398	116	44532588
WT_60D_1	198856	891500	130	44483406
KO_45D_1	157244	809532	100	44607016
KO_60D_3	209530	1686102	120	45430982
KO_60D_2	209488	4251802	100	44618186
KO_45D_2	198394	909118	92	44466288
WT_60D_3	192948	1216288	106	44164550

4 Reads Align

4.1 Align to the reference genome

After getting clean reads, we used HISAT to align the clean reads to the reference genome. The alignment results are shown as follows.

Sample Name	Total Clean Read	Total Mapping Genome Ratio	Uniquely Mapping Genome Ratio
KO_45D_1	44.61	89.36	84.77
KO_45D_2	44.47	87.93	83.58

KO_45D_3	42.53	87.90	83.58
KO_60D_1	44.53	87.60	82.94
KO_60D_2	44.62	86.05	81.03
KO_60D_3	45.43	86.77	82.01
WT_45D_1	44.66	88.86	84.33
WT_45D_2	43.90	89.09	84.44
WT_45D_3	45.42	87.32	82.83
WT_60D_1	44.48	87.72	83.40
WT_60D_2	44.33	87.63	83.18
WT_60D_3	44.16	87.02	82.63

4.2 Align to the reference gene

Align clean reads to the reference genes by using Bowtie2 to get the alignment result.

Sample Name	Total Clean Read	Total Mapping Gene Ratio	Uniquely Mapping Gene Ratio
KO_45D_1	44.61	74.74	71.34
KO_45D_2	44.47	75.34	71.85
KO_45D_3	42.53	74.56	71.20
KO_60D_1	44.53	68.33	64.18
KO_60D_2	44.62	61.96	57.83
KO_60D_3	45.43	65.31	61.12
WT_45D_1	44.66	71.54	67.60
WT_45D_2	43.90	71.55	67.62
WT_45D_3	45.42	71.12	67.22
WT_60D_1	44.48	64.35	59.65
WT_60D_2	44.33	63.68	58.88
WT_60D_3	44.16	61.64	57.01

5 Significant DEGs

The statistical results of significant DEGS are as follows.

Diff Plan	Diff Num
WT60D-vs-KO60D	8475
WT45D-vs-WT60D	10168
WT45D-vs-KO45D	10712
KO45D-vs-KO60D	10476
