# Supplementary material for: Ranolazine rescues the heart failure phenotype of PLN-deficient human pluripotent stem cell-derived cardiomyocytes
Source: Stem Cell Reports. 2022 Mar 24;17(4):804–19. doi: 10.1016/j.stemcr.2022.02.016 (PMC9023809; doi:10.1016/j.stemcr.2022.02.016)
Supplement: Document S2. Article plus supplemental information [file mmc2.pdf]

Stem Cell Reports
Article
OPEN ACCESS

Ranolazine rescues the heart failure phenotype of *PLN*-deficient human pluripotent stem cell-derived cardiomyocytes

Youxu Jiang,[1,5] Xiaowei Li,[2,5] Tianwei Guo,[1,5] Wen-Jing Lu,[1] Shuhong Ma,[1] Yun Chang,[1,4] Yuanxiu Song,[4] Siyao Zhang,[1] Rui Bai,[1] Hongyue Wang,[1] Man Qi,[3] Hongfeng Jiang,[1,*] Hongjia Zhang,[1,*] and Feng Lan[3,*]
[1]Beijing Laboratory for Cardiovascular Precision Medicine, MOE Key Laboratory of Biomedical Engineering for Cardiovascular Disease Research, Anzhen Hospital, Capital Medical University, Beijing Institute of Heart, Lung and Blood Vessel Diseases, Beijing 100029, China
[2]Department of Cardiology, The First Affiliated Hospital of Zhengzhou University, Zhengzhou 450052, China
[3]Fuwai Hospital Chinese Academy of Medical Sciences, Shenzhen, Shenzhen Key Laboratory of Cardiovascular Disease, State Key Laboratory of Cardiovascular Disease, Key Laboratory of Pluripotent Stem Cells in Cardiac Repair and Regeneration, Chinese Academy of Medical Sciences and Peking Union Medical College, Shenzhen, China
[4]Department of Cardiology, Peking University Third Hospital, Beijing, China
[5]These authors contributed equally
*Correspondence: hfjiang@ccmu.edu.cn (H.J.), zhanghongjia722@ccmu.edu.cn (H.Z.), fenglan@ccmu.edu.cn (F.L.)
https://doi.org/10.1016/j.stemcr.2022.02.016

SUMMARY

Phospholamban (PLN) is a key regulator that controls the function of the sarcoplasmic reticulum (SR) and is required for the regulation of cardiac contractile function. Although PLN-deficient mice demonstrated improved cardiac function, PLN loss in humans can result in dilated cardiomyopathy (DCM) or heart failure (HF). The CRISPR-Cas9 technology was used to create a PLN knockout human induced pluripotent stem cell (hiPSC) line in this study. PLN deletion hiPSCs-CMs had enhanced contractility at day 30, but proceeded to a cardiac failure phenotype at day 60, with decreased contractility, mitochondrial damage, increased ROS production, cellular energy metabolism imbalance, and poor Ca^{2+} handling. Furthermore, adding ranolazine to PLN knockout hiPSCs-CMs at day 60 can partially restore Ca^{2+} handling disorders and cellular energy metabolism, alleviating the PLN knockout phenotype of HF, implying that the disorder of intracellular Ca^{2+} transport and the imbalance of cellular energy metabolism are the primary mechanisms for PLN deficiency pathogenesis.

INTRODUCTION

Heart failure (HF) is a clinical symptom that manifests itself in the late stages of different cardiovascular illnesses and is now the main cause of mortality from cardiovascular disorders (Joseph et al., 2017; Metra and Teerlink, 2017; Murphy et al., 2020). HF is characterized by abnormal Ca^{2+} handling. One of the major causes of improper calcium therapy is faulty sarcoplasmic reticulum (SR) function (Peana and Domeier, 2017). The cardiac sarcoplasmic/endoplasmic reticulum (ER) Ca^{2+}-dependent ATPase 2a (SERCA2a) mediates calcium absorption by the SR and initiates relaxation, whereas phospholamban (PLN) is a regulatory phosphoprotein of SERCA2a activity (Kranias and Hajjar, 2012).

PLN is a 52-amino acid protein that alternates between monomeric and pentameric forms (MacLennan et al., 1998). The binding of a PLN monomer suppresses the activity of SERCA2a, whereas the pentamer functions as an inactive reservoir that is unable to interact with SERCA2a. Because of the essential regulatory function of SERCA2a, PLN is a major regulator of SR function and cardiac contractility and may be an important target for therapy in heart disease (Asahi et al., 2003). The overexpression of PLN decreases myocardial SR Ca^{2+} uptake and lowers Ca^{2+} load and contractile characteristics (Pallikkuth et al., 2013). PLN ablation, however, resulted in considerably improved Ca^{2+} cycling and myocardial contractility (Law et al., 2018). Furthermore, mutations in human PLN (e.g., R9C, Arg14del) that cause SR function deficits are linked to dilated cardiomyopathy (Cuello et al., 2021; Haghighi et al., 2003; Medeiros et al., 2011; Schmitt et al., 2003), and it has been reported that a naturally occurring loss-of-function human PLN mutation (PLN null) leads to severe cardiac dilation and HF (Haghighi et al., 2003).The PLN-deficient animals, however, showed no obvious developmental or anatomical defects, but they did have improved adulthood myocardial performance without alterations in heart rate and hyperdynamic cardiac function (Lorenz and Kranias, 1997).

Human and mouse PLN-null cardiac phenotypes differ dramatically, which may be related to intrinsic variations in heart physiology and Ca^{2+}-cycling processes between mice and humans. This indicates that more precise human cardiac models are required to investigate the pathogenic mechanism of PLN deficiency in humans. As a result, we used CRISPR-Cas9 gene editing technology to knock out the *PLN* gene in human induced pluripotent stem cells (hiPSCs), then generated these $PLN^{-/-}$ hiPSCs into cardiomyocytes (hiPSCs-CMs) and assessed the morphological

 © 2022 The Authors.
This is an open access article under the CC BY license (http://creativecommons.org/licenses/by/4.0/).

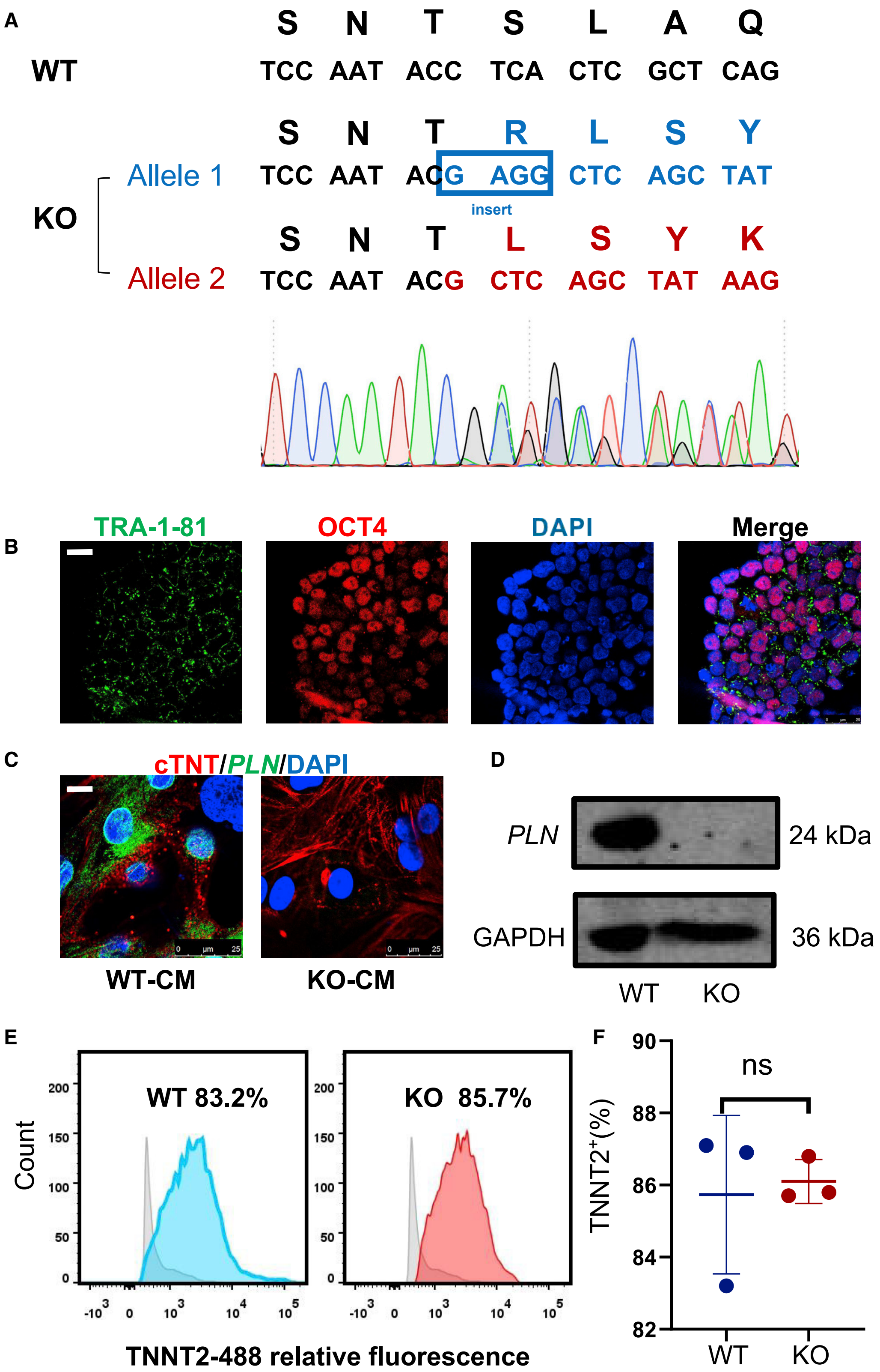

(legend on next page)

and functional alterations. The researchers discovered that PLN deletion hiPSCs-CMs had enhanced myocardial contractility at day 30, but they proceeded to a cardiac failure phenotype at day 60. Further research indicated that the processes causing HF include a malfunction in intracellular calcium ion transport and an imbalance in cellular energy consumption. Furthermore, adding the anti-arrhythmia medication ranolazine to PLN knockout hiPSCs-CMs at day 60 can partially repair cell energy metabolism and calcium transport abnormalities, relieving the PLN knockout (KO) phenotype of HF. These findings suggested that ranolazine might be a promising candidate medication for treating diseases caused by PLN dysfunction, which leads to SR function deficits.

RESULTS

Generation of homozygous PLN KO hiPSCs

An episomal vector-based CRISPR-Cas9 method was used to produce the PLN-KO hiPSC line (epiCRISPR). The guide RNA (gRNA) was engineered to target exon two of PLN, and the epiCRISPR containing the gRNA was electroporated into urine-derived hiPSCs (Xie et al., 2017). Following drug testing, the DNA of seven surviving clones was taken and enlarged for PCR screening verification. Among these clones was a *PLN* gene KO cell line with a seven-nucleotide (CTCACTC) deletion in one allele and a four-nucleotide (GAGG) insertion in another allele (Figure 1A), resulting in two frameshift coding sequences and *PLN* gene deletion. We also obtained the cell line of PLN heterozygote KO(+/−) (one allele 7-bp deletion in one allele, ACTTGCT) (Figure S1F). These $PLN^{-/-}$ hiPSCs had a normal morphology and karyotypes (Figure S1A). This cell line has the human pluripotency markers TRA-1-81 and OCT4 (Figure 1B). Furthermore, teratoma assays revealed that PLN-KO hiPSC preserved the ability to differentiate into three germ layers (Figure S1B).

PLN-deficient hiPSCs can differentiate into CMs

Because PLN primarily functions in the heart, PLN-KO hiPSCs were differentiated into CMs using small-molecule-based procedures (S1c). Immunocytochemistry revealed typical PLN localization in wild-type (WT) hiPSC-CMs, but not in PLN-KO hiPSC-CMs at day 30 (Figure 1C). On day 30, compared with the WT, western blot (WB) data indicated that PLN protein was not expressed in PLN-KO hiPSC-CMs (Figure 1D). The expression of PLN protein in heterozygous cells was 50.5% (Figure S1G). Flow cytometry was used to assess the effectiveness of CM differentiation, and the PLN-KO hiPSC-CMs displayed strong expression of the myocardial-specific marker cardiac troponin T (cTnT) (nearly 85%) at day 30 (Figures 1E and 1F), with no statistical difference when compared to the WT ($p = 0.79$).

PLN-KO causes a decrease in myocardial contractility

CM contraction is the most critical fundamental requirement for maintaining heart function (Penefsky, 1994). To assess the impact of PLN-KO on cardiac contractility, we evaluated the contractile force of hiPSC-CMs. The contractility amplitude of PLN-KO hiPSC-CM initially increased compared to WT at day 30, but it decreased with the extended cardiac cycle at day 60. The contractility amplitude of PLN heterozygous hiPSC-CMs increased slightly compared to WT at day 45 and started decreasing indistinctly at day 60 (Figures 2A–2D). After adding 500 nM isoprenaline (ISO), the contractility amplitude of PLN-KO hiPSC-CMs increased significantly at day 30, but the contractility amplitude increase in PLN-KO hiPSC-CMs with ISO began to decrease at day 45. The contraction amplitude of PLN-KO hiPSC-CMs did not increase with ISO at day 60 (Figures S2A–S2D).These findings showed that PLN deficit will eventually result in an HF phenotype in hiPSC-CMs, which was consistent with earlier findings of severe HF caused by PLN expression disorders in people.

PLN-deficient hiPSC-CMs recapitulate HF phenotypes *in vitro*

HF is a process of cardiac systolic function decompensation (Maack et al., 2019). We observe the morphological and functional alterations of PLN-KO hiPSC-CMs at various time points based on this. In human CMs, increased CM size is a key characteristic of HF (Harvey and Leinwand, 2011). Using flow cytometry, we discovered that on the 60th day, the size of PLN-KO hiPSC-CMs rose by 43.3% to WT (Figures 2E and 2F). On days 30, 45, and

Figure 1. Phospholamban (PLN) knockout (KO) does not affect the pluripotency of hiPSCs and differentiation into hiPSC-CMs
(A) Sequence chromatograms demonstrate a homozygous *PLN* gene KO line in which 1 allele deleted 7 nucleotides (CTCACTC) and another allele deleted 7 nucleotides (CTCACTC) and inserted 4 nucleotides (GAGG).
(B) Immunofluorescence staining of PLN-KO colonies for the pluripotency markers TRA-1-81 and OCT4. Scale bar, 25 μm.
(C) Representative immunofluorescence images showing the intracellular protein distribution of PLN and cardiac troponin T (cTnT) in WT and PLN-KO hiPSC-CMs at day 30. Nuclei were counterstained with DAPI. Scale bars, 25 μm.
(D) Western blot analysis of PLN in WT and PLN-KO hiPSC-CMs at day 15.
(E and F) Flow cytometry analysis for TNNT2 from representative WT and PLN-KO differentiation protocols before purification at day 15. The results are presented as means ± SEMs of 3 independent experiments. ns, not significant, unpaired 2-sided Student's t test.

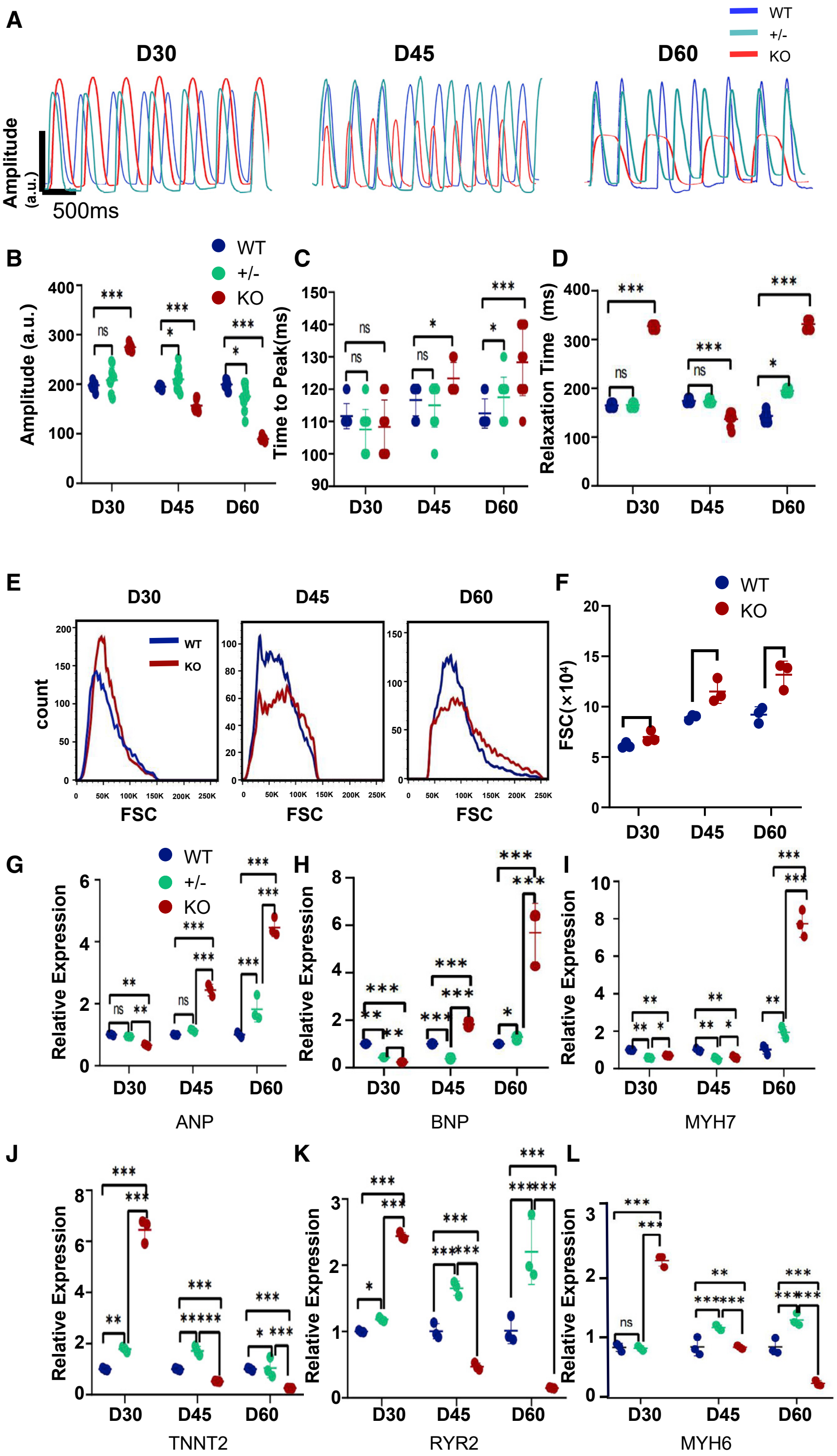

Figure 2. PLN-deficient hiPSC-CMs recapitulate a decrease in myocardial contractility and HF phenotypes *in vitro*

(A) Representative line scan images in WT, PLN heterozygotes, and PLN-KO hiPSC-CMs myocardial contractility at days 30, 45, and 60.

(B–D) Quantification of amplitude, time to peak, and relaxation time in WT and PLN-KO hiPSC-CMs (n = 12 cells per group).

(E and F) Calibration of forward scatter (FSC; 10,000 cells per sample, n = 3) showing an increased cellular size beginning 45 days post-cardiac differentiation in PLN-KO hiPSC-CMs.

(G–L) Quantitative real-time-PCR analysis of HF-related genes in WT, PLN heterozygotes, and PLN-KO hiPSC-CMs at days 30, 45, and 60. The results are presented as means ± SEMs of 3 independent experiments.

*$p < 0.05$; **$p < 0.01$; ***$p < 0.001$; ns, not significant, unpaired 2-sided Student's t test.

60, we examined key cardiac functional markers (Gaggin and Januzzi, 2013; Magrì et al., 2020; Waldmüller et al., 2011) (*ANP/BNP/MYH7/TNNT/RYR2/MYH6*) in PLN-KO hiPSC-CMs. On day 60, the expression of *ANP/BNP/MYH7* PLN-KO hiPSC-CMs increased compared to WT (Figures 2G–2I), whereas the expression of *TNNT2/RYR2/MYH6 PLN*-KO hiPSC-CMs decreased (Figures 2J–2L). At day 60, *PLN*-KO hiPSC-CMs displayed typical HF phenotypes,

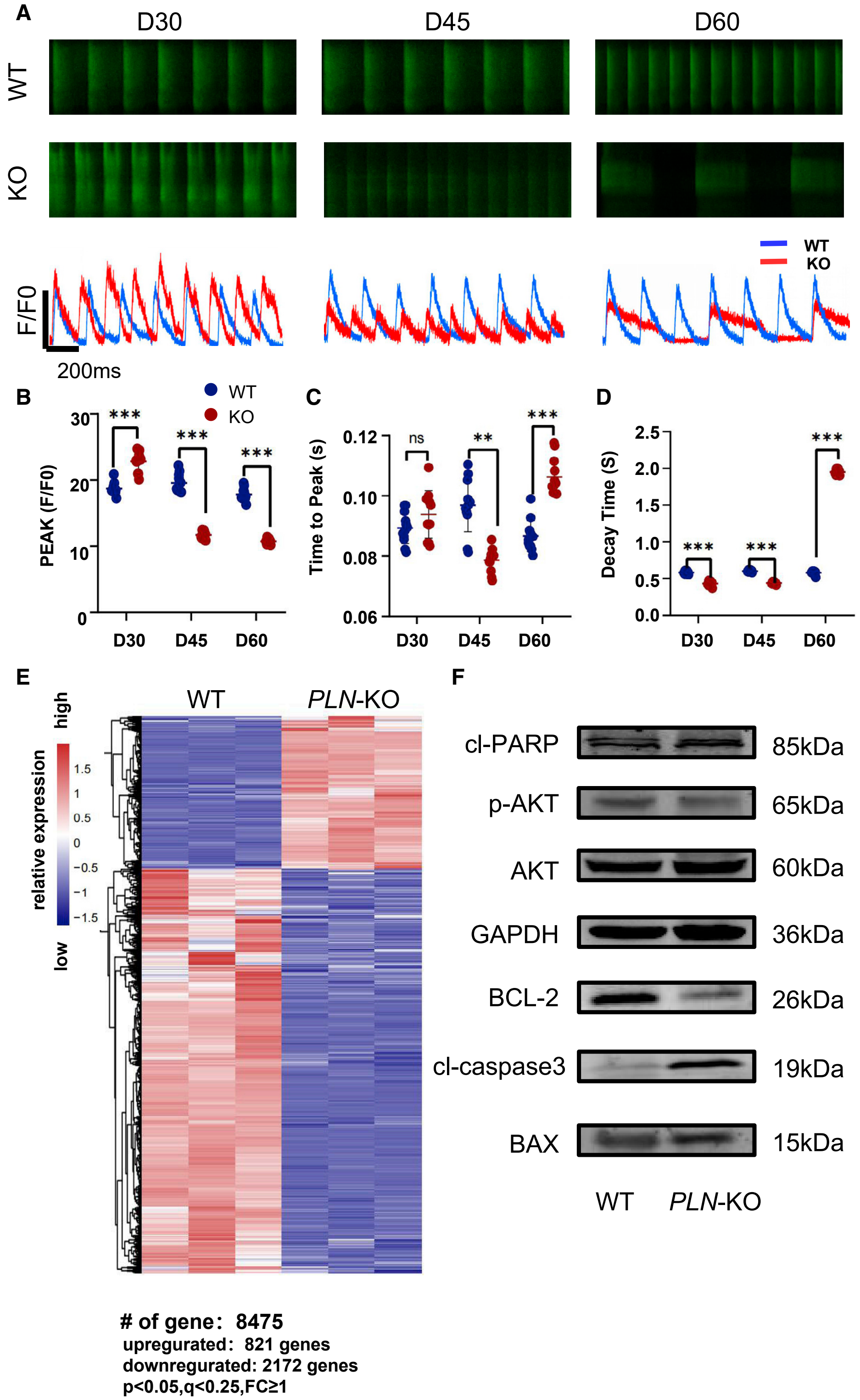

Figure 3. PLN deficiency leads to abnormal cardiac calcium transients and promotes the activation of apoptotic pathway in PLN-KO hiPSC-CMs

(A) Representative line scan images in WT-GCaMP and PLN-KO-GCaMP hiPSC-CMs at days 30, 45, and 60.

(B–D) Quantification of peak, time to peak, and calcium decay time in WT-GCaMP and PLN-KO-GCaMP hiPSC-CMs (n = 12 cells per group).

(legend continued on next page)

according to our findings. Consistently, major cardiac functional assays revealed that PLN heterozygous hiPSC-CMs showed mild phenotypical changes with a trend similar to that of PLN-KO hiPSC-CMs, but remarkably delayed (Figures 2G–2L). Combined with the insignificant change in the difference in myocardial contractility of PLN heterozygotes compared to WT, to better discover the role of *PLN*, we used the CMs of *PLN*-KO hiPSC-CMs for further investigation.

PLN deficiency leads to abnormal cardiac calcium transients in PLN-KO hiPSC-CMs

Calcium transport in CMs is one of the most essential processes for controlling CM contraction and relaxation (Cartwright et al., 2005). Calcium transport dysfunction can result in serious HF. The calcium sensor green fluorescent calcium-modulated protein 6 fast type (GCaMP6f) was inserted into the adeno-associated virus integration site 1 (AAVS1) locus of PLN-KO cell lines to detect alterations in calcium transients (Li et al., 2019) (Figure S1E). On day 30, the PLN-KO hiPSC-CMs showed a substantial increase in Ca^{2+} reuptake rate and Ca^{2+} release amplitude when compared to WT. The Ca^{2+} reuptake rate and release rate of PLN-KO hiPSC-CMs increased at day 45, whereas the Ca^{2+} release amplitude began to decrease. The Ca^{2+} absorption rate and release amplitude of PLN-KO hiPSC-CMs were considerably lower than those of WT, and the calcium transport cycle was much longer at day 60 (Figures 3A–3C).Consistently, after adding 500 nM ISO, the Ca^{2+} release amplitude of PLN-KO hiPSC-CMs increased significantly at day 30, Ca^{2+} release amplitude of PLN-KO hiPSC-CMs with ISO did not increase significantly at day 60 (Figures S2E–S2H). These results suggested that PLN-KO hiPSC-CMs showed evident calcium transport problems.

PLN-KO activates oxidative stress and promotes the activation of the apoptotic pathway

Because PLN is a critical regulatory factor of Ca^{2+} recovery by SERCA2a in SR (Kranias and Hajjar, 2012), we found that the control of Ca^{2+} in CMs is disrupted after PLN deletion, leading to the HF phenotype. We also looked at whether PLN-KO affected other key pathways in hiPSC-CM. We performed a global transcriptome analysis comparing PLN-KO and WT human CMs to establish these pathways at day 60. As *PLN* was deleted, 821 genes were upregulated and 2,172 genes were downregulated when compared to controls (Figure 3E). The results of gene set enrichment analysis (GSEA) revealed that pathways regulating mitochondrial function, cellular proliferation, and cardiac muscle contraction, among others, were significantly enriched and downregulated with PLN deletion (Figure S3C), whereas oxidative stress and apoptosis were significantly increased in DCM.

Reactive oxygen species (ROS)-induced myocardial apoptosis is an important physiological mechanism that leads to HF (Zhang et al., 2012). The expression of ROS, B cell lymphoma-2 (BCL-2)-associated X protein (BAX), and BCL-2 in myocardial cells was examined next to determine the amount of myocardial cell damage following PLN-KO hiPSC-CMs. Flow cytometry was used to initially assess changes in cellular ROS levels. On day 30 and day 45, there was no significant change in PLN-KO hiPSC-CMs ROS, whereas PLN-KO hiPSC-CMs ROS increased considerably on day 60. (Figures 4A and 4B). Phosphatidylinositol 3-kinase (PI3K) activation increases AKT phosphorylation (p-AKT) and activates downstream signaling. It is essential for the circulatory system, myocardial apoptosis, and metabolism (Cantoni et al., 2012; Siman et al., 2015; Yang et al., 2019). The expression levels of p-AKT/BAX/cl-caspase3/cl-PARP (poly(ADP-ribose) polymerase) were enhanced in the PLN-KO hiPSC-CMs group ($p < 0.05$) as measured by AKT/p-AKT/caspase3 pathway WB analysis on day 60 (Figures 3F and S3). The PLN-KO hiPSC-CMs group had substantially reduced BCL-2 expression ($p < 0.05$). These findings clearly suggested that PLN, in addition to being necessary for appropriate calcium handling via the control of SERCA2a expression and activity, has a larger role in CM function. PLN-KO may produce oxidative stress in cells, resulting in myocardial apoptosis.

PLN-deficient CMs develop mitochondrial dysfunction

Mitochondria are the most essential organelles for sustaining CM energy metabolism, and Ca^{2+} excess damage can cause mitochondrial malfunction, which will trigger numerous, particularly oxidative, stress damages (Kumar et al., 2019; Santulli et al., 2015). Previous findings from this work show that Ca^{2+} transport disruption and oxidative stress damage emerge after PLN deletion; therefore, alterations in mitochondria after PLN-KO must be investigated. As a result, the number of *ND1*, *ND2*, and housekeeping *ACTB* in PLN-KO hiPSC-CMs was measured by comparing the number of *ND1*, *ND2*, and housekeeping

(E) Gene clustering by *Z* score using Euclidean distance metric in WT and PLN-KO samples at day 60. Gene expression fold change (FC) was calculated by PLN-KO over control. $p < 0.05$, $q < 0.25$, FC $\geq$ 1.

(F) Immunoblot analysis of AKT apoptotic pathway signaling (cl-RAPR, AKT, phosphorylated AKT,BCL-2,cl-caspase3, and BAX) in WT and PLN-KO hiPSC-CMs at day 60. Results are presented as means ± SEMs of 3 independent experiments.

*$p < 0.05$; **$p < 0.01$; ***$p < 0.001$; ns, not significant, unpaired 2-sided Student's t test.

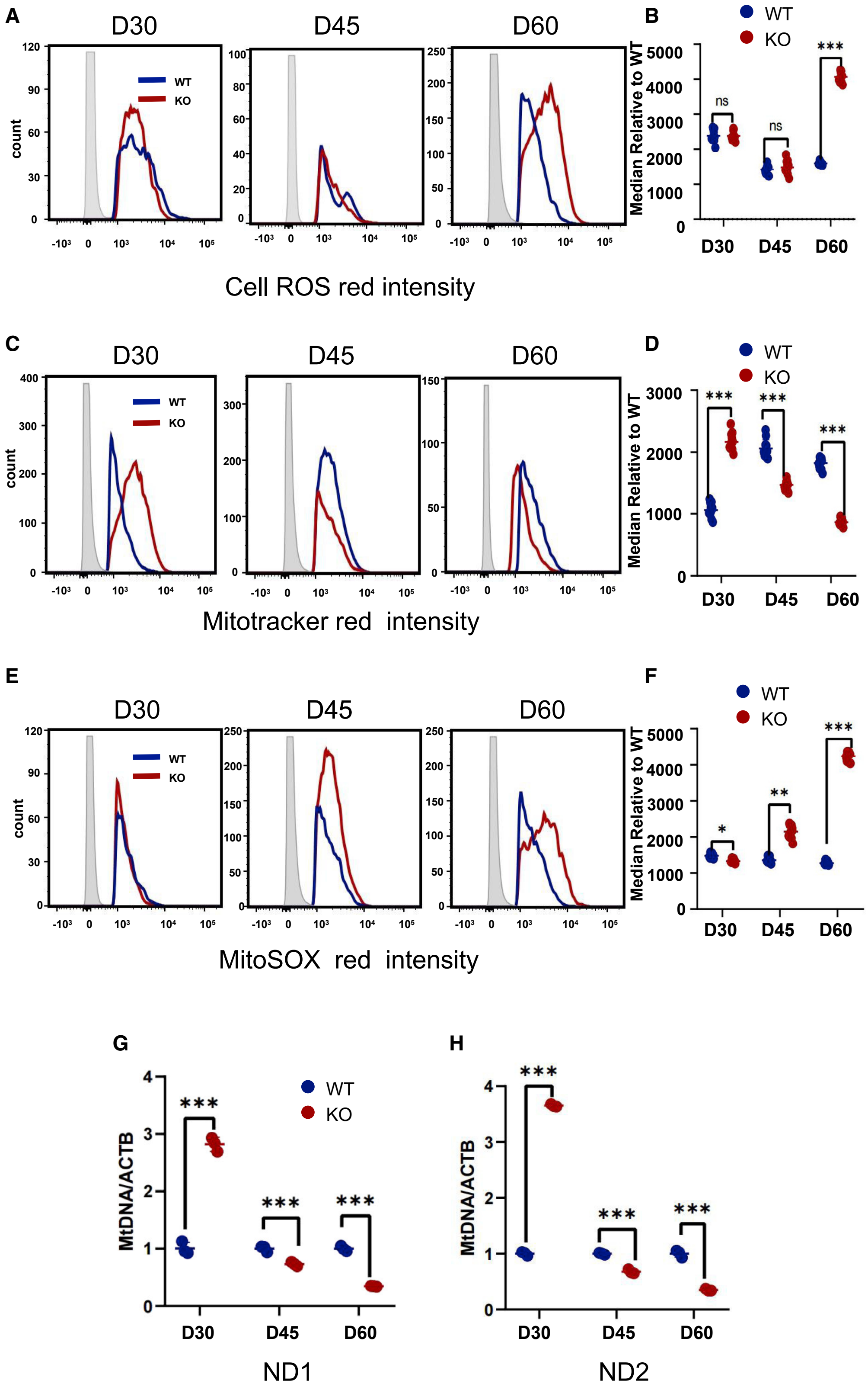

(legend on next page)

ACTB at 30, 45, and 60 days. The expression of mitochondrial *ND1/ND2* increased in PLN-KO hiPSC-CMs on day 30 but decreased on day 60 (Figures 4G and 4H).

To further understand the impact of PLN loss on metabolism, flow cytometry was used to measure mitochondrial content, total cellular ROS, and mitochondrial-specific ROS on days 30, 45, and 60 (Figures 4C–4F). On day 30, mitochondrial content increased compared to WT, while mitochondrial SOX remained the same. MitoTracker levels began to fall after day 45, whereas mitochondrial SOX levels continued to rise. MitoTracker dropped substantially at day 60, but mitochondrial SOX rose dramatically. These findings revealed that following PLN-knockdown, early mitochondrial function was hyperactive, and Ca^{2+} transport began to develop abnormalities over time, leading to increased mitochondrial damage and reduced mitochondrial content.

PLN-KO induced energy metabolism disorders

CM contraction and relaxation is an active, energy-consuming activity (Fink et al., 2017). Previous Gene Ontology (GO) and Kyoto Encyclopedia of Genes and Genomes pathway (KEGG) enrichment analyses have shown a reduction in mitochondrial functioning and number, but the characteristics of energy metabolism remain unclear. *PPARA*, *PPARGC1A*, and *PPARGC1B* (Figures 5D–5F), which are involved in the fatty acid oxidation of PLN-KO hiPSC-CMs, began to decline at day 30, while genes involved in glucose utilization, such as *GNPNAT1*, *PGM3*, and *GFPT1*, were upregulated (Figures 5A–5C), indicating that energy metabolism disorders began to occur. The glucose metabolism and levels of indicators related to the fatty acid metabolism of PLN-KO hiPSC-CMs had considerably reduced by day 60. Basal oxygen consumption rates (OCRs), capacity for maximum cellular respiration, and ATP level (Figures 5G–5J, S5A, and S5B) rose fast in the PLN-KO hiPSC-CMs at day 30 but declined at day 60 in the WT. All of these findings indicate that the energy metabolism of PLN-KO hiPSC-CMs is extremely low and that the energy metabolism is clearly dysfunctional.

Improvement in energy metabolism and calcium transport disorders by ranolazine can rescue the HF phenotype of PLN-KO hiPSC-CMs

Ranolazine is a well-tolerated medication that selectively inhibits the late sodium current (Cattaneo et al., 2015), which acts as an enhancer of the outward mode of the sodium-calcium exchanger (NCX) by blocking late sodium currents; thus, it indirectly promotes Ca^{2+} efflux, while being a type of fatty acid oxidation inhibition. It has beneficial metabolic properties and has no effect on heart rate or blood pressure (Coppini et al., 2013). In PLN-KO hiPSC-CMs, energy metabolism and calcium transport abnormalities are identified, which are one of the pathways leading to mitochondrial damage, cellular ROS, and death. Based on this, we treated PLN-KO hiPSC-CMs with ranolazine to determine whether it improved the HF phenotype. We discovered that after continuously treating PLN-KO hiPSC-CMs with a therapeutic dose of ranolazine (1 μm) for 5 days, the Ca^{2+} transient abnormality was considerably decreased, and the maximal Ca^{2+} amplitude was increased from day 30 (Figures 6A–6D and S4). After ranolazine therapy, the contractility amplitude of PLN-KO hiPSC-CMs recovered to approximately 75% of the WT level (Figures 6E–6H). We also accessed the effects of ranolazine on PLN heterozygous hiPSC-CMs. The chronic administration of β-adrenergic agonists, such as isoproterenol (Wang et al., 2014), has been shown to aggravate hypertrophic cardiomyopathy (HCM) and induce HF in HCM models of disease (Frey et al., 2004). When treated with 1 μM ISO for 1 week, the calcium release amplitudes increased in WT hiPSC-CMs and significantly decreased in PLN heterozygous hiPSC-CMs(+/−) at day 60 (Figures S6A–S6D). After a therapeutic dose of ranolazine (1 μm) for 5 days, the maximal Ca^{2+} amplitude of PLN heterozygous hiPSC-CM added with ISO was partially recovered (Figures S6E–S6J).

At the same time, energy metabolism is significantly restored, glucose metabolism and fatty acid metabolism are significantly improved at day 60 (Figures S5E–S5J), the basal and maximum respiration of PLN-KO hiPSC-CMs are significantly improved from day 30 (Figures 7J and S5A—S5D), and the ATP level of PLN-KO hiPSC-CMs is significantly restored (Figure 7G). Flow cytometry findings

Figure 4. PLN-deficient hiPSC-CMs develop mitochondrial dysfunction and cells activate oxidative stress

(A and B) Quantification of Cell ROS Red intensity obtained by flow cytometry demonstrates a significantly reduced fluorescence intensity in PLN-KO hiPSC-CMs at days 30, 45, and 60 as compared with WT hiPSC-CMs (n = 12, 10,000 cells per sample).

(C and D) Quantification of MitoTracker Red intensity obtained by flow cytometry demonstrates a significantly reduced fluorescence intensity in PLN-KO hiPSC-CMs at days 30, 45, and 60 as compared with WT hiPSC-CMs (n = 12).

(E and F) Quantification of MitoSOX Red intensity obtained by flow cytometry demonstrates a significantly reduced fluorescence intensity in PLN-KO hiPSC-CMs at days 30, 45, and 60 as compared with WT hiPSC-CMs (n = 12).

(G and H) qPCR analysis of mitochondrial DNA (*ND1* and *ND2*) to nuclear DNA (β-actin) ratio at days 30, 45, and 60 of cardiac differentiation (n = 3, 10,000 cells per sample). The results are presented as means ± SEMs of 3 independent experiments.

*p < 0.05; **p < 0.01; ***p < 0.001; ns, not significant, unpaired 2-sided Student's t test.

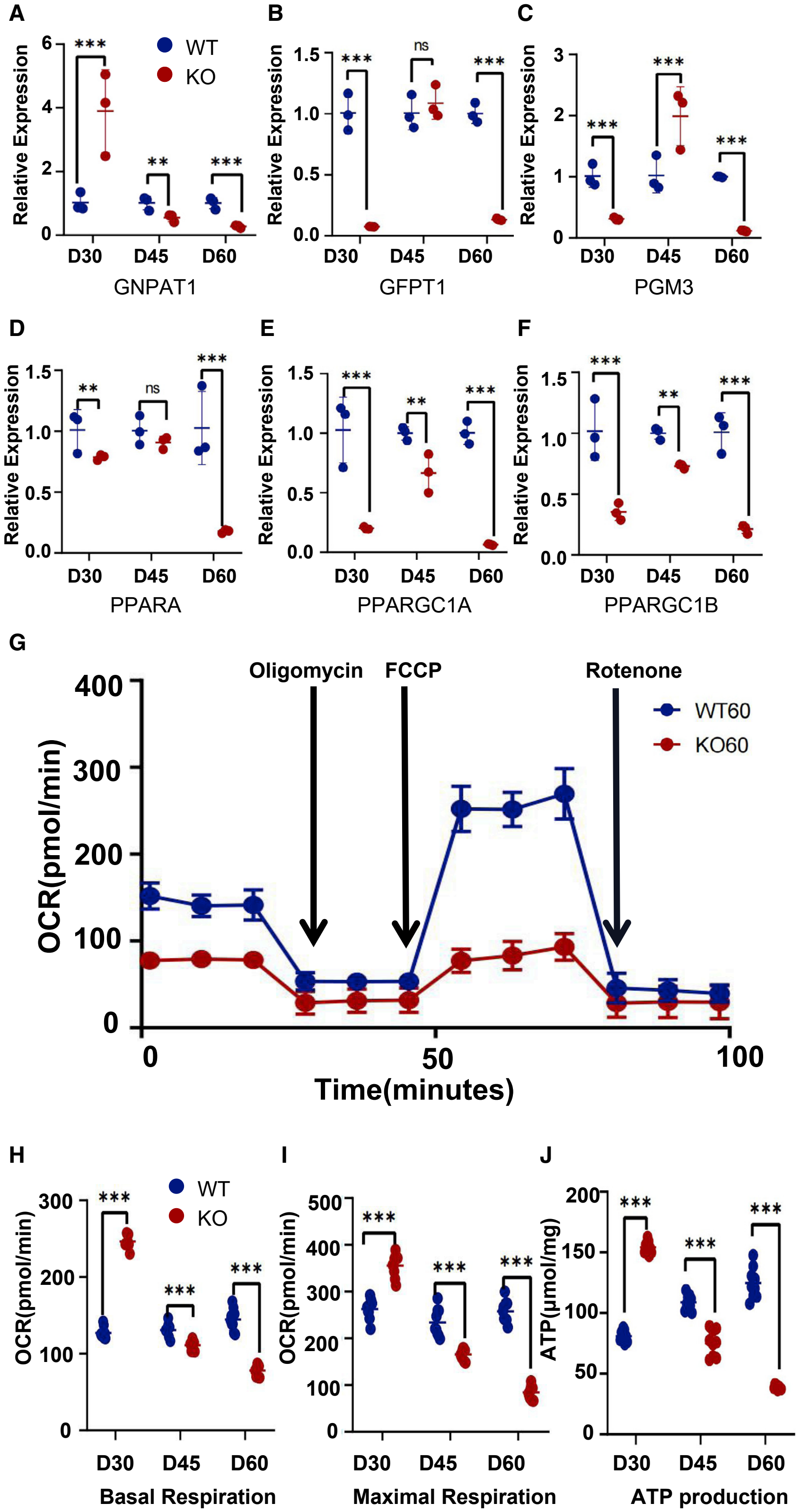

Figure 5. PLN-KO induced energy metabolism disorders

(A–C) Quantitative real-time-PCR analysis of glucose utilization-related genes in WT and PLN-KO hiPSC-CMs at days 30, 45, and 60 (n = 3).

(D–F) Quantitative real-time-PCR analysis of fatty acid oxidation-related genes in WT and PLN-KO hiPSC-CMs at days 30, 45, and 60 (n = 3).

(G) Assessment of the oxygen consumption rate (OCR) was performed with the application of compounds to assess ATP production (oligomycin), maximum (Max) respiration (FCCP, carbonyl cyanide-4 [trifluoromethoxy] phenylhydrazone), and halted respiration (antimycin A and rotenone) for WT and PLN-KO hiPSC-CMs at day 60 (n = 9).

(H–J) Basal respiration of WT and PLN-KO hiPSC-CMs at days 30, 45, and 60 (H); (I) maximum respiration of WT and PLN-KO hiPSC-CMs at days 30, 45, and 60; (J) the ATP level in WT and PLN-KO hiPSC-CMs at days 30, 45, 60 (n = 9). The results are presented as means ± SEMs of 3 independent experiments. $^{*}p < 0.05$; $^{**}p < 0.01$; $^{***}p < 0.001$; ns, not significant, unpaired 2-sided Student's t test.

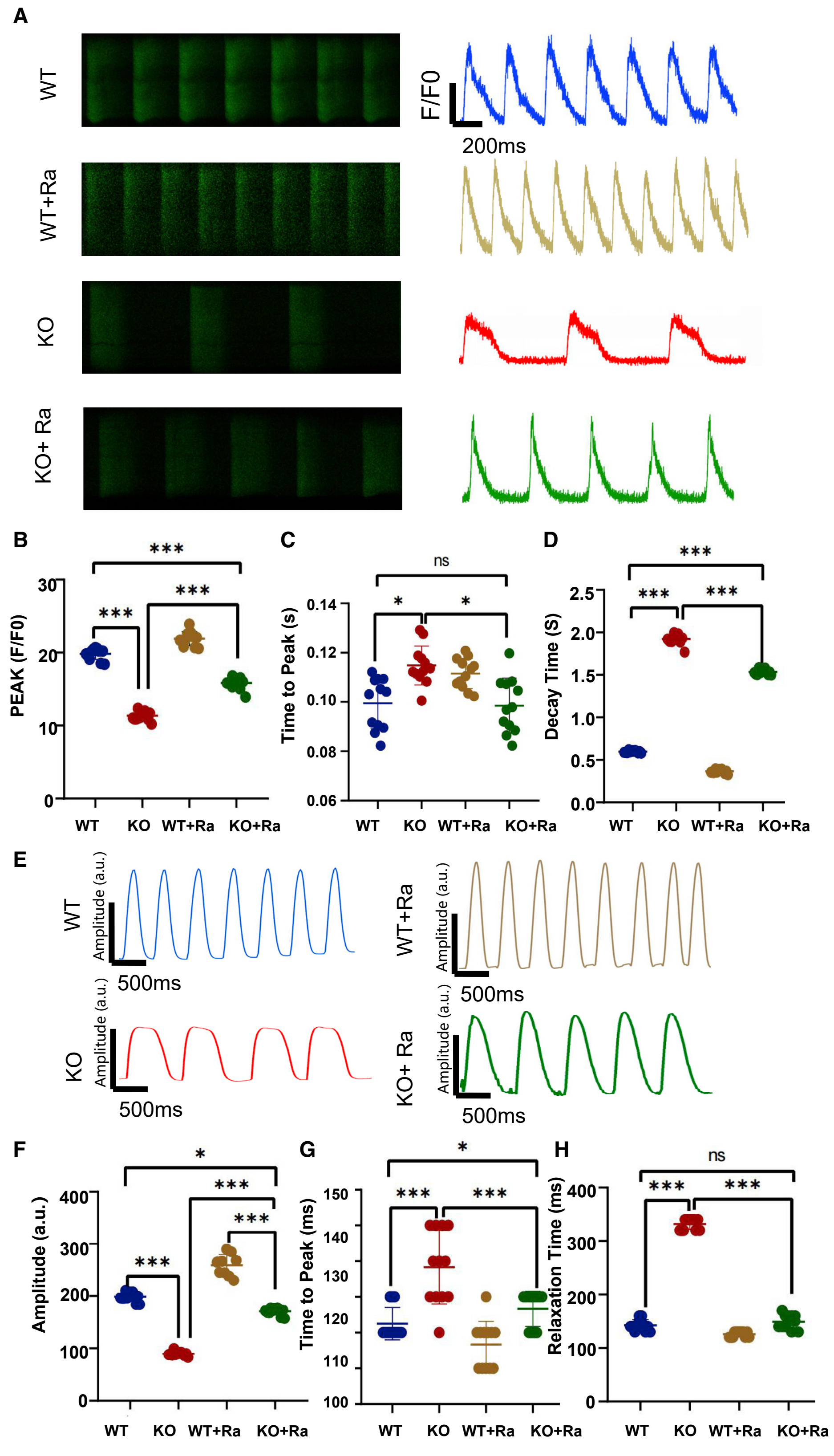

(legend on next page)

showed that mitochondrial MitoTracker recovered considerably to WT levels (Figures 7A and 7D), whereas mitochondrial SOX (Figures 7B and 7E) and cellular ROS (Figures 7C and 7F) were dramatically decreased. The qPCR findings showed that the *ANP/BNP/RYR2/TNNT2* levels of the ranolazine treatment group fell substantially (Figure 7I). The findings suggest that ranolazine therapy can ameliorate the HF phenotype of PLN-KO hiPSC-CMs. The WB and qPCR results following ranolazine therapy (Figures 7H and S3B) demonstrated that ranolazine administration increased AKT phosphorylation, as shown by a substantial increase in the p-AKT level. We also discovered that BAX expression reduced in PLN-KO hiPSC-CMs and BCL-2 expression rose. AKT is a downstream kinase that is controlled by PI3K, and an elevated p-AKT:AKT ratio indicates that the PI3K-AKT pathway is active, perhaps boosting the recruitment of myocardial protective factors. These findings demonstrated that ranolazine can ameliorate the HF phenotype of the PLN-KO hiPSC-CMs.

DISCUSSION

A PLN-KO hiPSC-CMs model was created in this study, which has the potential to be exploited in the research of PLN-related illnesses. Previous research on the *PLN* gene has primarily focused on animal models; however, the conclusions based on animal models contradict the findings of PLN-related research in humans. In fact, an article in 2003 reported that two patients with homozygous mutation of the PLN 39th amino acid mutation as stop codon underwent heart transplantation at the ages of 16 and 27, respectively, due to severe cardiac dilation and HF (Haghighi et al., 2003). The authors found that the expression of PLN mRNA decreased by 50% in one of the recipient hearts, and the expression of *PLN* protein was not detected. These two patients may be considered to be PLN null cases, which is similar to the PLN-KO hiPSC model of our study. Our study looked at the functional changes and processes that occurred following PLN deletion in hiPSC-CMs. Early in the PLN-KO mice model, SR Ca^{2+} uptake increased considerably, accompanied by enhanced contractility of CMs, which is consistent with the rise in contractility of the PLN-KO animal model. The contractility of PLN-KO hiPSC-CM, however, diminished with time and finally acquired HF traits. On day 45, PLN-KO hiPSC-CMs showed a decrease in Ca^{2+} transport capacity, which was earlier than the start of the HF phenotype, suggesting that Ca^{2+} transport impairment is one of the causes of HF following PLN-KO. Interestingly, we observed that ISO reduced decay time for the PLN-KO CM from day 30. This is not expected since PLN is the major contributor to the ISO-mediated increase in SR Ca^{2+} reuptake. It has been well documented that the iPSC-CMs are immature. In addition to the PLN-SERCA2a pathway, extracellular calcium can be transient through the T-type calcium channel on cell membranes (Karakikes et al., 2015). ISO can enhance the activity of the T-type calcium channel and reduce the disorder of calcium transient (Li et al., 2018). Numerous investigations have revealed that energy metabolism disorders are one of the key causes of HF (Taegtmeyer and Dietze, 1999). The normal state of cardiac cells is dominated by fatty acid metabolism, whereas energy metabolism problems result from mitochondrial dysfunction (Leung, 2004). The number of mitochondria increased and ATP synthesis increased but fatty acid metabolism decreased at the early stage of PLN-KO hiPSC-CMs (day 30), indicating that the enhancement of ATP synthesis consumed to maintain myocardial contractile and calcium transport was based on enhanced glycolysis of anaerobic metabolism in myocardial cells. Meanwhile, the energy metabolism function of the PLN-KO myocardial cells was defective. With the continued increase in metabolic diseases, the supply of ATP cannot keep up with the rising demand for calcium transport. On day 45, glycolysis, ATP generation, and calcium transport performance all began to deteriorate, resulting in mitochondrial damage and an increase in cellular ROS. Mitochondrial damage and cellular ROS generation exacerbated cell energy metabolism problems and improper calcium transport. Ca^{2+} transport levels fell considerably at day 60, as shown in the present study, as did the number of mitochondria and ATP levels, resulting in a negative feedback cycle of calcium transport failure and mitochondrial

Figure 6. Ranolazine recovered calcium transport disorders and myocardial contractility in PLN-KO hiPSC-CMs at day 60

(A) Representative line scan images in WT-GCaMP, PLN KO-GCaMP, WT-GCaMP + ranolazine, and PLN-KO-GCaMP + ranolazine hiPSC-CMs at day 60.

(B–D) Quantification of peak, time to peak, and calcium decay time in WT-GCaMP, PLN KO-GCaMP, WT + ranolazine, and PLN-KO-GCaMP + ranolazine hiPSC-CMs at day 60 (n = 12 cells per group).

(E) Representative line scan images in WT-GCaMP, PLN-KO-GCaMP, WT + ranolazine, and PLN-KO-GCaMP + ranolazine hiPSC-CM myocardial contractility at day 60.

(F–H) Quantification of amplitude, time to peak, and relaxation time in WT, PLN-KO,WT + ranolazine, and PLN-KO + ranolazine hiPSC-CMs at day 60 (n = 12 cells per group). The results are presented as means ± SEMs of 3 independent experiments.

$^{*}p < 0.05$; $^{**}p < 0.01$; $^{***}p < 0.001$; ns, not significant, unpaired 2-sided Student's t test. One-way ANOVA and least significant difference (LSD) test were used to compare the parameters between groups.

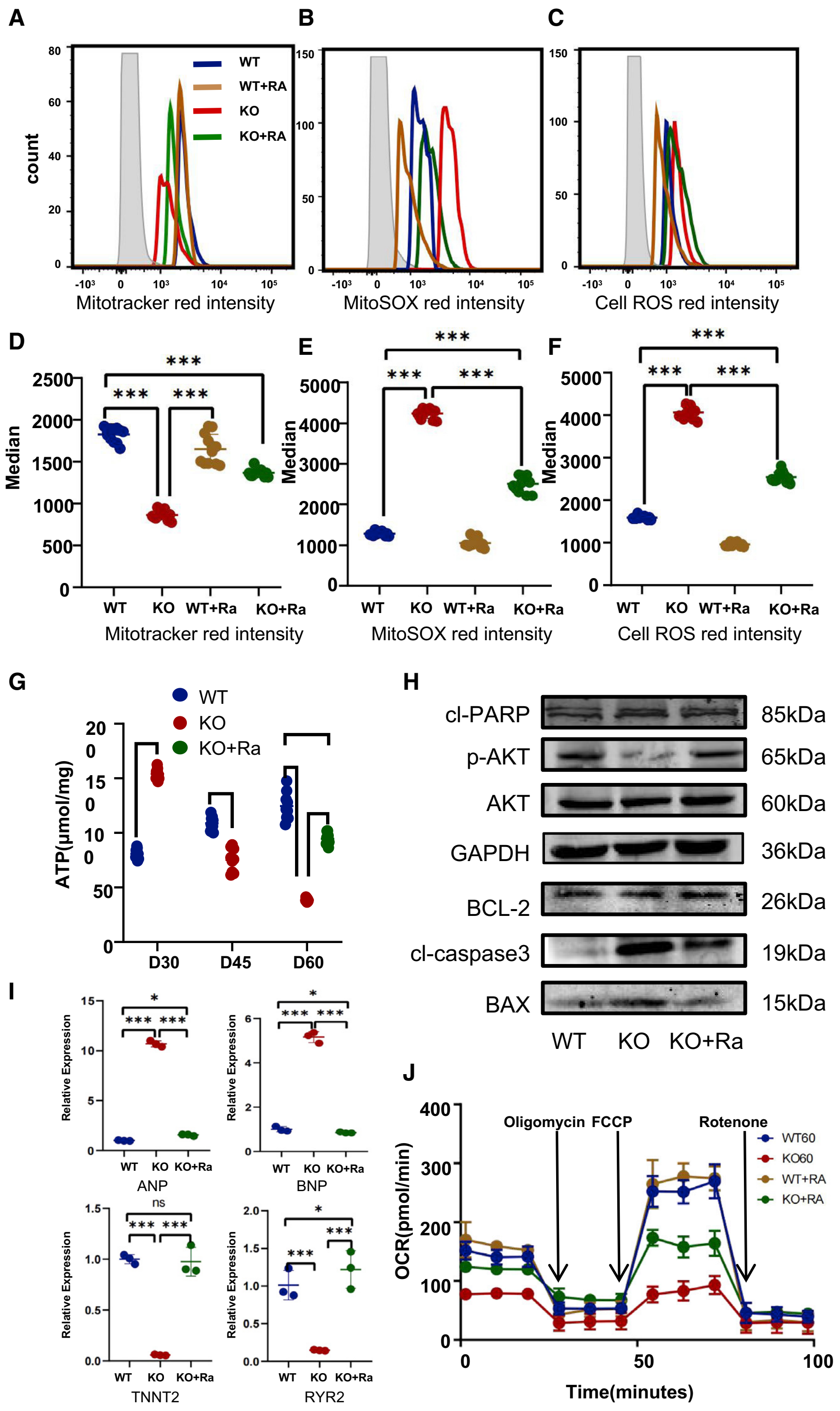

Figure 7. Ranolazine recovered mitochondrial dysfunction and cells activate oxidative stress in PLN-KO hiPSC-CMs at day 60

(A and D) Quantification of MitoTracker Red intensity obtained by flow cytometry fluorescence intensity in WT, PLN-KO, WT + ranolazine, and PLN-KO + ranolazine hiPSC-CMs at day 60 (n = 12).

(B and E) Quantification of MitoSOX Red intensity obtained by flow cytometry fluorescence intensity in WT, PLN-KO, WT + ranolazine, and PLN-KO + ranolazine hiPSC-CMs at day 60 (n = 12).

(C and F) Quantification of Cell ROS Red intensity obtained by flow cytometry fluorescence intensity in WT, PLN-KO, WT + ranolazine, and PLN-KO + ranolazine hiPSC-CMs at day 60 (n = 12) .

(G) The ATP level in WT, PLN-KO and PLN-KO + ranolazine hiPSC-CMs at day 60 (n = 9).

(H) Immunoblot analysis of AKT apoptotic pathway signaling (cl-RAPR, AKT, phosphorylated AKT, BCL-2, cl-caspase3, and BAX) in WT, PLN-KO, and PLN-KO + ranolazine hiPSC-CMs at day 60.

(I) Quantitative real-time-PCR analysis of HF-related genes in WT, PLN-KO, and PLN-KO + ranolazine hiPSC-CMs at day 60.

(J) Assessment of OCR in WT, PLN-KO, WT + ranolazine, and PLN-KO + ranolazine hiPSC-CMs at day 60 (n = 9). The results are presented as means ± SEMs of 3 independent experiments.

$*p < 0.05$; $**p < 0.01$; $***p < 0.001$; ns, not significant, unpaired 2-sided Student's t test. One-way ANOVA and LSD test were used to compare the parameters between groups.

damage. WB data reveal that at day 60, the total quantity of AKT in PLN-KO hiPSC-CMs did not differ substantially from the WT, but the phosphorylation level of ATK in PLN-KO hiPSC-CMs dropped, inhibiting the level of downstream BCL-2. Simultaneously, the expression of BAX was substantially elevated, triggering caspase3/PARP pathway-dependent apoptosis. These modifications account for the development of HF phenotypes in *PLN*-KO hiPSC-CMs.

Ranolazine has recently been found to be effective in enhancing myocardial diastolic function and treating

diastolic HF (Wang et al., 2019). The fundamental mechanisms of action, however, remain unknown. In this study, it was discovered that by adding ranolazine to *PLN*-KO hiPSC-CMs with an HF phenotype, the glucose metabolism level was restored, Ca^{2+} transport was restored, mitochondrial damage and the number of mitochondria were restored, the level of ROS was decreased, and the phenotype of HF was recovered.

As a late sodium current inhibitor, ranolazine can discharge calcium in the cytoplasm outside the cell via NCX channels on the cell membrane, decreasing mitochondrial and cellular ROS damage caused by Ca^{2+} excess in the cytoplasm, and resulting in a partially corrected calcium metabolism imbalance (Rayner-Hartley and Sedlak, 2016). Cardiovascular metabolism, however, uses oxygen to oxidize fatty acids or glucose to create energy. Under normal physiological circumstances, CMs increase oxidation capacity mostly through the utilization of fatty acids rather than glucose (Fosslien, 2003). Ranolazine, as a pFOX (partial fatty acid oxidase) inhibitor, can reduce fatty acid oxidation, enhance glucose oxidation efficiency, and boost ATP synthesis efficiency in CMs (Sabbah et al., 2002) under anoxic conditions, providing a certain energy foundation for repairing SR Ca^{2+} transport barriers. In this study, PLN-KO hiPSC-CMs showed a decrease in both fatty acid and glucose metabolism, a significant decrease in the production of ATP, and obvious defects in calcium transient at day 60. After adding ranolazine, glycolysis oxidation of PNL-KO hiPSC-CMs increased at day 60; we also found that ranolazine can reduce the calcium overload (Mosqueira et al., 2018) and then reduce the mitochondrial damage. Due to the sufficient supply of oxygen content in this experiment, our results suggest that after using ranolazine, the oxygen consumption rate of WT cells did not change significantly, while the basic OCR and maximum OCR of PLN-KO CMs recovered at day 60. At the same time, the marker of fatty acid metabolism rebounded, indicating that it did not significantly inhibit fatty acid oxidation. These results show that both fatty acid and glycolysis oxidation of PLN-KO hiPSC-CMs can recover to normal levels by ranolazine treatment, which partially rescues the phenotype of PLN-KO hiPSC-CMs. This study also found that in the HF phenotypes produced by PLN-KO, AKT phosphorylation reduced, leading to the activation of the apoptotic protein caspase3/RAPR pathway and, as a result, apparent apoptosis. It can suppress the production of the apoptotic protein caspase3/PARP after ranolazine therapy by increasing the phosphorylation of AKT, therefore decreasing cell death. We speculated that ranolazine may rescue the phenotype by alleviating the calcium overload. By reducing the damage to mitochondria caused by calcium overload, the function of mitochondria was restored partially, the fatty acid oxidation of PLN-KO hiPSC-CMs was improved, and the level of PLN-KO hiPSC-CMs cells metabolism was restored to the level of WT.

In conclusion, our work found that PLN deficiency can result in HF phenotypes in PLN-KO hiPSC-CMs. One probable explanation is that calcium transport dysfunction in PLN-KO hiPSC-CMs causes energy metabolism dysfunction, mitochondrial damage, and cell death. Simultaneously, this study used a pharmacological approach to repair the HF phenotypes induced by PLN deficiency. Ranolazine can enhance calcium transport and energy metabolism, giving researchers a new target and scientific foundation for treating HF caused by PLN insufficiency.

EXPERIMENTAL PROCEDURES

Cell culture

This study was approved by the Ethics Committee of Anzhen Hospital, Capital Medical University. Human induced pluripotent stem cell (hiPSC) lines were cultured on feeder-free Matrigel (Corning, USA) in E8 medium (Cellapy, China) under a humidified 5% CO_2 atmosphere at 37°C. To the medium, 0.5 mM of EDTA (HyClone, USA) was added for cell passage when the cell confluency reached 70%–80%.

In vitro CM differentiation and purification

A chemically defined molecular-based method was used for CM differentiation (Burridge et al., 2014). Spontaneous contracting could be observed 7–8 days after differentiation. Then, the CMs were purified by using a metabolic-selection method using the purification medium composed of RPMI 1640 without glucose, 213 μg/mL of L-ascorbic acid 2-phosphate, 500 μg/mL of *Oryza sativa*-derived recombinant human albumin, and 5 mM of sodium DL-lactate (Sigma, USA).

Genome editing

The sgRNA targeting for exon 2 of PLN (TTCTTATAGCTGAGCGAGTG) was designed by the CRISPR design tool (http://crispr.mit.edu/). The gRNA was cloned into an epiCRISPR-Cas9 vector. We dissociated 1 × 10^5 hiPSC 0.5 mmol/L EDTA, to which 5 μg of the plasmid was electrotransferred into cells using the 4D nucleus effector system (Lonza, Germany). The cells were then seeded onto 6-well plates coated with Matrigel and cultured in the E8 medium. After drug screening, the remaining colonies were transferred into a 48-well plate and investigated by DNA sequencing primer sequences of PLN listed in table below.

Name	Forward sequence (5′–3′)	Reverse sequence (5′–3′)
PLN	CTGAGGATAGGTTACATAGATG	AGGTTGTAGCAGAACTTCA

The generation of hiPSC-GCaMP and $PLN^{-/-}$-GCaMP is as follows: 2.5 μg AAVS1_sgRNA plasmid (Addgene, #100554, USA) and 2.5 μg

pAAVS1-PC-GCaMP6f plasmid (Addgene, #73503) were electroporated together into hiPSC and $PLN^{-/-}$ hiPSCs. The methods for puromycin screening (S1D), selection, and identification of clones are as described above (Li et al., 2019).

Immunostaining and imaging analyses

The cells were fixed in 4% paraformaldehyde (PFA), permeabilized in 0.5% Triton X-100 (Sigma, USA) for 15 min, and blocked with 3% BSA (Sigma) at room temperature for 30 min. Later, the cells were incubated with the primary antibody overnight at 4°C and incubated with a secondary antibody at 37°C for 1 h. After washing 3 times with PBS for 5 min each, the cells were counterstained with 300 nM DAPI (Invitrogen, USA) for 5 min (antibodies listed in Table S2). The samples were then imaged using the Leica DMI 4000B confocal microscope (Leica, Germany).

Flow cytometry

The cells were digested with 0.5 mM EDTA. The single-cell suspension was blocked with 3% BSA and incubated with the primary antibody for 30 min. After washing 3 times with PBS, the cells were incubated with the secondary antibody for 30 min.

For the quantification of mitochondria and ROS, dissociated hiPSC-CMs were stained with 50 nM of MitoTracker Red (C1049B; Beyotime, China), 10 μM Cell ROS Red (C10491; Invitrogen), and 2.5 μM of MitoSOX Red (M36008; Invitrogen). Live cells were incubated with these dyes (made in RPMI 1640; Corning) for 20 min at 37°C and under a 5% CO_2 atmosphere. The samples were analyzed by fluorescence-activated cell sorting (FACS) analysis (BD Biosciences, USA). The results were analyzed by using FlowJo X software.

Ca^{2+} imaging

PLN-KO-GCaMP hiPSC-CMs were seeded onto confocal dishes. Intracellular calcium flow was imaged at 40× by confocal microscopy (tcs5 SP5; Leica). Spontaneous Ca^{2+} transients were collected at a sampling rate of 1 ms/line using a line scan mode at 37°C and under a 5% CO_2 atmosphere. The results were analyzed by using ImageJ and Igor.

The transcript expression experiment (RNA sequencing [RNA-seq])

The qualified library was sequenced using bgiseq platform (project no. f20ftsccwlj6851_howkse). Gene differential expression analysis was performed using deseq2. The screening of differential genes is based mainly on the differential multiple (fold change value) and q value (p adjusted value, corrected p value). The screening criteria of significantly different genes were $|\log2 \text{ fold change}| \geq 1$ and $q < 0.05$. All of the differential genes were analyzed by hierarchical cluster analysis using R-package, and the heatmap was drawn. The differential genes were counted by GO, and the significantly enriched GO items were found according to the standard of $q < 0.05$.

RNA extraction and quantitative real-time PCR

The cells (2×10^5) were extracted with TRIzol (Life Technologies, USA) and DNase I (Life Technologies) to eliminate DNA contamination. Then, 1 μg RNA was reverse transcribed into cDNAs using the PrimeScript Reverse Transcription System (Takara, Japan). The program of quantitative real-time-PCR was performed on Icycler iQ5 (Bio-Rad, USA) using the 2× SYBR Master Mix (Takara, Japan). The primers sequences used are listed in Table S1.

WB

The cells were digested and centrifuged, and then the cells were resuspended using a protein extraction reagent (Thermo Fisher, #78501, USA) added with a phosphatase inhibitor mixture (Thermo Fisher, #1862495). The samples were incubated on ice for 30 min, shaken every 10 min, and then centrifuged at 12,000 rpm for 15 min. The BCA method was used to measure the protein concentration. The same amount of protein was resolved by gel electrophoresis and then transferred onto the polyvinylidene fluoride (PVDF) membrane. The membrane was blocked with 5% skim milk powder at 37°C for 1 h and then incubated with the primary and secondary antibodies listed in Table S2.

Detection of myocardial contractility

CMs were seeded into a 6-well culture plate pre-coated with matrix glue. Videos were captured under the Leica DMI 4000B. The video of the beating of myocardial cells was filmed for 3–5 s, saved in the original .czi format, and then converted to an uncompressed .avi format (70 frames per second [fps]). A special plug-in (MUSCLEMOTION) for video analysis was installed in ImageJ, and the results were analyzed and stored (Fosslien, 2003; Grune et al., 2019).

Detection of ATP content in cells

Lysate (200 μL) was added into each well of the 6-well plate to lyse the cells. After lysis, the cells were centrifuged at 12,000 × *g*/4°C for 5 min, and the supernatant was collected. Next, 100 μL of the ATP working solution was added into the 96-well culture plate at room temperature for 3–5 min. Two seconds after adding 20 μL of the sample or prototype, the relative light unit (RLU) value was measured by a luminometer or liquid scintillation meter to detect the ATP content in cells. The concentration of ATP in the sample was calculated according to the standard curve.

Data analysis and statistics

Data represent the mean ± standard error of the mean (SEM). Statistical significance was evaluated by a Student's t test and ANOVA was used for the comparison of multiple effects. $p < 0.05$ was considered to be statistically significant.

Data and code availability

We uploaded the RNA-seq raw data to the National Center for Biotechnology Information (NCBI) archives series (accession no.: PRJNA763655, https://www.ncbi.nlm.nih.gov/bioproject/PRJNA763655).

SUPPLEMENTAL INFORMATION

Supplemental information can be found online at https://doi.org/10.1016/j.stemcr.2022.02.016.

AUTHOR CONTRIBUTIONS

F.L., H.J., and H.Z. conceived the idea and designed the experiments. Y.J., X.L., and T.G. performed the cell experiments and data analysis. Y.J. and T.G. prepared the manuscript. S.M. and Y.S. are responsible for the cell culture experiments and the collection and assembly of the data. Y.C., S.Z., and M.Q. contributed to the molecular experiments. Y.J. and H.W. contributed to the function analysis. Y.W. and R.B. helped with the revisions. All of the authors read and approved the final manuscript.

CONFLICTS OF INTEREST

The authors declare no competing interests.

ACKNOWLEDGMENTS

We gratefully acknowledge funding support from Beijing Natural Science Foundation no. Z190013, National Natural Science Foundation of China no. 81970205, the non-profit Central Research Institute Fund of Chinese Academy of Medical Sciences (2019PT320026, 2021-F02), and the Shenzhen Fundamental Research Program (ZDSYS20200923172000001).

Received: October 9, 2021
Revised: February 22, 2022
Accepted: February 22, 2022
Published: March 24, 2022

REFERENCES

Asahi, M., Sugita, Y., Kurzydlowski, K., De Leon, S., Tada, M., Toyoshima, C., and MacLennan, D.H. (2003). Sarcolipin regulates sarco(endo)plasmic reticulum Ca2+-ATPase (SERCA) by binding to transmembrane helices alone or in association with phospholamban. Proc. Natl. Acad. Sci. U S A *100*, 5040–5045.

Burridge, P.W., Matsa, E., Shukla, P., Lin, Z.C., Churko, J.M., Ebert, A.D., Lan, F., Diecke, S., Huber, B., Mordwinkin, N.M., et al. (2014). Chemically defined generation of human cardiomyocytes. Nat. Methods *11*, 855–860.

Cantoni, S., Cavallini, C., Bianchi, F., Bonavita, F., Vaccari, V., Olivi, E., Frascari, I., Tassinari, R., Valente, S., Lionetti, V., et al. (2012). Rosuvastatin elicits KDR-dependent vasculogenic response of human placental stem cells through PI3K/AKT pathway. Pharmacol. Res. *65*, 275–284.

Cartwright, E.J., Schuh, K., and Neyses, L. (2005). Calcium transport in cardiovascular health and disease–the sarcolemmal calcium pump enters the stage. J. Mol. Cell. Cardiol. *39*, 403–406.

Cattaneo, M., Porretta, A.P., and Gallino, A. (2015). Ranolazine: drug overview and possible role in primary microvascular angina management. Int. J. Cardiol. *181*, 376–381.

Coppini, R., Ferrantini, C., Yao, L., Fan, P., Del Lungo, M., Stillitano, F., Sartiani, L., Tosi, B., Suffredini, S., Tesi, C., et al. (2013). Late sodium current inhibition reverses electromechanical dysfunction in human hypertrophic cardiomyopathy. Circulation *127*, 575–584.

Cuello, F., Knaust, A.E., Saleem, U., Loos, M., Raabe, J., Mosqueira, D., Laufer, S., Schweizer, M., van der Kraak, P., Flenner, F., et al. (2021). Impairment of the ER/mitochondria compartment in human cardiomyocytes with *PLN* p.Arg14del mutation. EMBO Mol. Med. *13*, e13074.

Fink, B.D., Bai, F., Yu, L., and Sivitz, W.I. (2017). Regulation of ATP production: dependence on calcium concentration and respiratory state. Am. J. Physiol. Cell Physiol. *313*, 146–c153.

Fosslien, E. (2003). Review: mitochondrial medicine–cardiomyopathy caused by defective oxidative phosphorylation. Ann. Clin. Lab. Sci. *33*, 371–395.

Frey, N., Barrientos, T., Shelton, J.M., Frank, D., Rütten, H., Gehring, D., Kuhn, C., Lutz, M., Rothermel, B., Bassel-Duby, R., et al. (2004). Mice lacking calsarcin-1 are sensitized to calcineurin signaling and show accelerated cardiomyopathy in response to pathological biomechanical stress. Nat. Med. *10*, 1336–1343.

Gaggin, H.K., and Januzzi, J.L., Jr. (2013). Biomarkers and diagnostics in heart failure. Biochim. Biophys. Acta *1832*, 2442–2450.

Grune, T., Ott, C., Häseli, S., Höhn, A., and Jung, T. (2019). The "MYOCYTER" - convert cellular and cardiac contractions into numbers with ImageJ. Scientific Rep. *9*, 15112.

Haghighi, K., Kolokathis, F., Pater, L., Lynch, R.A., Asahi, M., Gramolini, A.O., Fan, G.C., Tsiapras, D., Hahn, H.S., Adamopoulos, S., et al. (2003). Human phospholamban null results in lethal dilated cardiomyopathy revealing a critical difference between mouse and human. J. Clin. Invest. *111*, 869–876.

Harvey, P.A., and Leinwand, L.A. (2011). The cell biology of disease: cellular mechanisms of cardiomyopathy. J. Cel. Biol. *194*, 355–365.

Joseph, P., Leong, D., McKee, M., Anand, S.S., Schwalm, J.D., Teo, K., Mente, A., and Yusuf, S. (2017). Reducing the global burden of cardiovascular disease, Part 1: the epidemiology and risk factors. Circ. Res. *121*, 677–694.

Karakikes, I., Ameen, M., Termglinchan, V., and Wu, J.C. (2015). Human induced pluripotent stem cell-derived cardiomyocytes: insights into molecular, cellular, and functional phenotypes. Circ. Res. *117*, 80–88.

Kranias, E.G., and Hajjar, R.J. (2012). Modulation of cardiac contractility by the phospholamban/SERCA2a regulatome. Circ. Res. *110*, 1646–1660.

Kumar, A.A., Kelly, D.P., and Chirinos, J.A. (2019). Mitochondrial dysfunction in heart failure with preserved ejection fraction. Circulation *139*, 1435–1450.

Law, M.L., Prins, K.W., Olander, M.E., and Metzger, J.M. (2018). Exacerbation of dystrophic cardiomyopathy by phospholamban deficiency mediated chronically increased cardiac Ca(2+) cycling in vivo. Am. J. Physiol. Heart Circ. Physiol. *315*, 1544–h1552.

Leung, K. (2004). [11C]Acetoacetate. 2008 Oct 18 [Updated 2008 Nov 12]. In Molecular Imaging and Contrast Agent Database (MICAD) [Internet] (National Center for Biotechnology Information (US)). https://www.ncbi.nlm.nih.gov/books/NBK23622/.

Li, X., Lu, W.J., Li, Y., Wu, F., Bai, R., Ma, S., Dong, T., Zhang, H., Lee, A.S., Wang, Y., et al. (2019). MLP-deficient human pluripotent stem cell derived cardiomyocytes develop hypertrophic cardiomyopathy and heart failure phenotypes due to abnormal calcium handling. Cell Death Dis. *10*, 610.

Li, Y., Zhang, X., Zhang, C., Zhang, X., Li, Y., Qi, Z., Szeto, C., Tang, M., Peng, Y., Molkentin, J.D., et al. (2018). Increasing T-type

calcium channel activity by β-adrenergic stimulation contributes to β-adrenergic regulation of heart rates. J. Physiol. *596*, 1137–1151.

Lorenz, J.N., and Kranias, E.G. (1997). Regulatory effects of phospholamban on cardiac function in intact mice. Am. J. Physiol. *273*, H2826–H2831.

Maack, C., Eschenhagen, T., Hamdani, N., Heinzel, F.R., Lyon, A.R., Manstein, D.J., Metzger, J., Papp, Z., Tocchetti, C.G., Yilmaz, M.B., et al. (2019). Treatments targeting inotropy. Eur. Heart J. *40*, 3626–3644.

MacLennan, D.H., Kimura, Y., and Toyofuku, T. (1998). Sites of regulatory interaction between calcium ATPases and phospholamban. Ann. N Y Acad. Sci. *853*, 31–42.

Magrì, D., Mastromarino, V., Gallo, G., Zachara, E., Re, F., Agostoni, P., Giordano, D., Rubattu, S., Forte, M., Cotugno, M., et al. (2020). Risk stratification in hypertrophic cardiomyopathy. Insights from genetic analysis and cardiopulmonary exercise testing. J. Clin. Med. *9*, 1636.

Medeiros, A., Biagi, D.G., Sobreira, T.J., de Oliveira, P.S., Negrão, C.E., Mansur, A.J., Krieger, J.E., Brum, P.C., and Pereira, A.C. (2011). Mutations in the human phospholamban gene in patients with heart failure. Am. Heart J. *162*, 1088–1095.e81.

Metra, M., and Teerlink, J.R. (2017). Heart failure. Lancet (London, England) *390*, 1981–1995.

Mosqueira, D., Mannhardt, I., Bhagwan, J.R., Lis-Slimak, K., Katili, P., Scott, E., Hassan, M., Prondzynski, M., Harmer, S.C., Tinker, A., et al. (2018). CRISPR/Cas9 editing in human pluripotent stem cell-cardiomyocytes highlights arrhythmias, hypocontractility, and energy depletion as potential therapeutic targets for hypertrophic cardiomyopathy. Eur. Heart J. *39*, 3879–3892.

Murphy, S.P., Ibrahim, N.E., and Januzzi, J.L., Jr. (2020). Heart failure with reduced ejection fraction: a review. Jama *324*, 488–504.

Pallikkuth, S., Blackwell, D.J., Hu, Z., Hou, Z., Zieman, D.T., Svensson, B., Thomas, D.D., and Robia, S.L. (2013). Phosphorylated phospholamban stabilizes a compact conformation of the cardiac calcium-ATPase. Biophysical J. *105*, 1812–1821.

Peana, D., and Domeier, T.L. (2017). Cardiomyocyte Ca(2+) homeostasis as a therapeutic target in heart failure with reduced and preserved ejection fraction. Curr. Opin. Pharmacol. *33*, 17–26.

Penefsky, Z.J. (1994). The determinants of contractility in the heart. Comp. Biochem. Physiol. Physiol. *109*, 1–22.

Rayner-Hartley, E., and Sedlak, T. (2016). Ranolazine: a contemporary review. J. Am. Heart Assoc. *5*, e003196.

Sabbah, H.N., Chandler, M.P., Mishima, T., Suzuki, G., Chaudhry, P., Nass, O., Biesiadecki, B.J., Blackburn, B., Wolff, A., and Stanley, W.C. (2002). Ranolazine, a partial fatty acid oxidation (pFOX) inhibitor, improves left ventricular function in dogs with chronic heart failure. J. Card. Fail. *8*, 416–422.

Santulli, G., Xie, W., Reiken, S.R., and Marks, A.R. (2015). Mitochondrial calcium overload is a key determinant in heart failure. Proc. Natl. Acad. Sci. U S A *112*, 11389–11394.

Schmitt, J.P., Kamisago, M., Asahi, M., Li, G.H., Ahmad, F., Mende, U., Kranias, E.G., MacLennan, D.H., Seidman, J.G., and Seidman, C.E. (2003). Dilated cardiomyopathy and heart failure caused by a mutation in phospholamban. Science *299*, 1410–1413.

Siman, F.D., Silveira, E.A., Fernandes, A.A., Stefanon, I., Vassallo, D.V., and Padilha, A.S. (2015). Ouabain induces nitric oxide release by a PI3K/Akt-dependent pathway in isolated aortic rings from rats with heart failure. J. Cardiovasc. Pharmacol. *65*, 28–38.

Taegtmeyer, H., and Dietze, G.J. (1999). Perspective: from increased energy metabolism to cardiac hypertrophy and failure: mediators and molecular mechanisms. Am. J. Cardiol. *83*, 1h–2h.

Waldmüller, S., Erdmann, J., Binner, P., Gelbrich, G., Pankuweit, S., Geier, C., Timmermann, B., Haremza, J., Perrot, A., Scheer, S., et al. (2011). Novel correlations between the genotype and the phenotype of hypertrophic and dilated cardiomyopathy: results from the German competence network heart failure. Eur. J. Heart Fail. *13*, 1185–1192.

Wang, G.T., Li, H., Yu, Z.Q., and He, X.N. (2019). Effects of ranolazine on cardiac function in rats with heart failure. Eur. Rev. Med. Pharmacol. Sci. *23*, 9625–9632.

Wang, Y., Liang, P., Lan, F., Wu, H., Lisowski, L., Gu, M., Hu, S., Kay, M.A., Urnov, F.D., Shinnawi, R., et al. (2014). Genome editing of isogenic human induced pluripotent stem cells recapitulates long QT phenotype for drug testing. J. Am. Coll. Cardiol. *64*, 451–459.

Xie, Y., Wang, D., Lan, F., Wei, G., Ni, T., Chai, R., Liu, D., Hu, S., Li, M., Li, D., et al. (2017). An episomal vector-based CRISPR/Cas9 system for highly efficient gene knockout in human pluripotent stem cells. Sci. Rep. *7*, 2320.

Yang, X., Zhao, T., Feng, L., Shi, Y., Jiang, J., Liang, S., Sun, B., Xu, Q., Duan, J., and Sun, Z. (2019). PM(2.5)-induced ADRB2 hypermethylation contributed to cardiac dysfunction through cardiomyocytes apoptosis via PI3K/Akt pathway. Environ. Int. *127*, 601–614.

Zhang, S., Liu, X., Bawa-Khalfe, T., Lu, L.S., Lyu, Y.L., Liu, L.F., and Yeh, E.T. (2012). Identification of the molecular basis of doxorubicin-induced cardiotoxicity. Nat. Med. *18*, 1639–1642.

Stem Cell Reports, Volume *17*

Supplemental Information

Ranolazine rescues the heart failure phenotype of *PLN*-deficient human pluripotent stem cell-derived cardiomyocytes

Youxu Jiang, Xiaowei Li, Tianwei Guo, Wen-Jing Lu, Shuhong Ma, Yun Chang, Yuanxiu Song, Siyao Zhang, Rui Bai, Hongyue Wang, Man Qi, Hongfeng Jiang, Hongjia Zhang, and Feng Lan

Supplementary Figure 1:Characterization of *PLN* KO hiPSCs and hiPSC-CM differentiation efficiency.Method details on the transcript GCaMP-expression of *PLN* KO hiPSCs

A 46 X, X normal karyotype of *PLN* KO hiPSCs

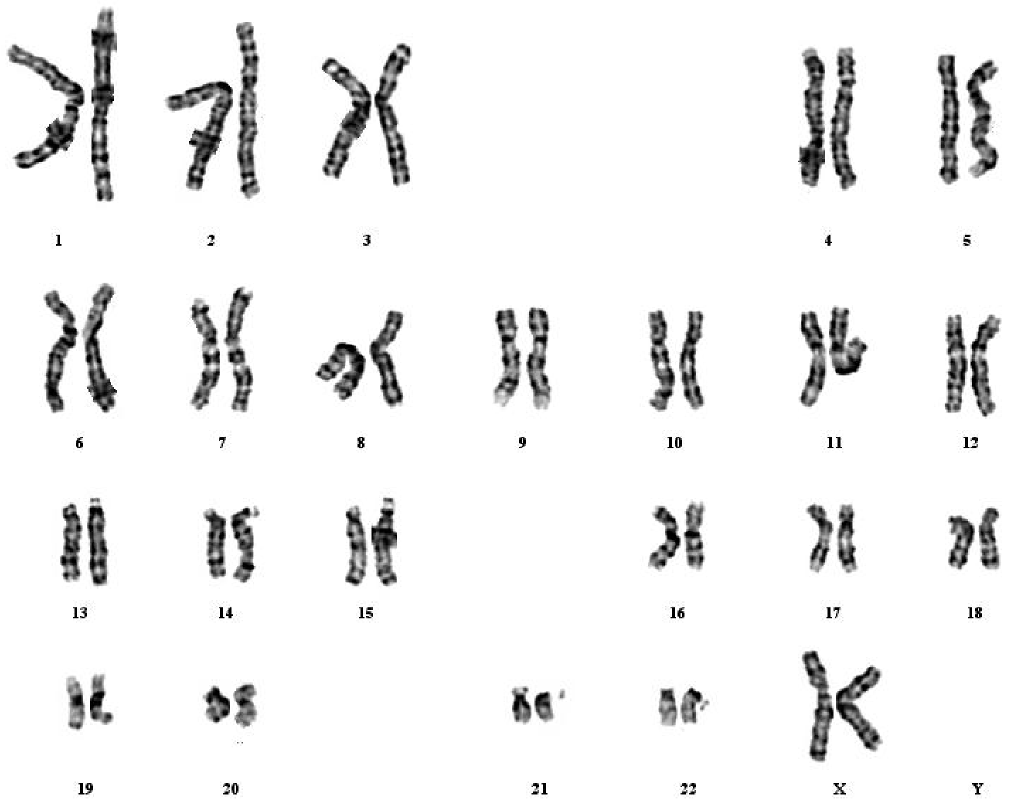

B Teratomas contain cell lineages from three germ layers (ectoderm, mesoderm and endoderm). Scale bars, 100μm.

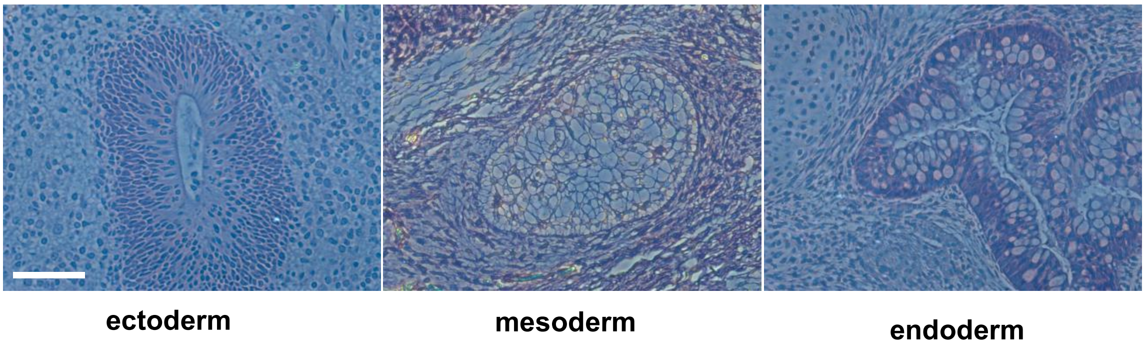

C Protocol for cardiac differentiation using small molecule-based methods

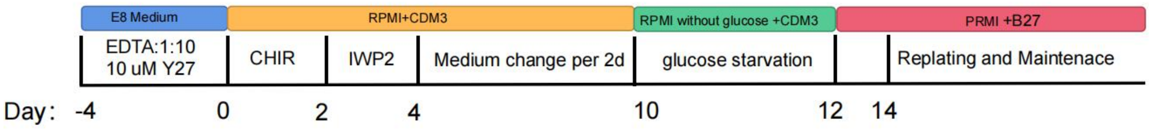

D:Schematic demonstrating the GCaMP-expression cassette that was integrated into AAVS1 of WT and *PLN* KO hiPSCs via nickase CRISPR/Cas9 editing.

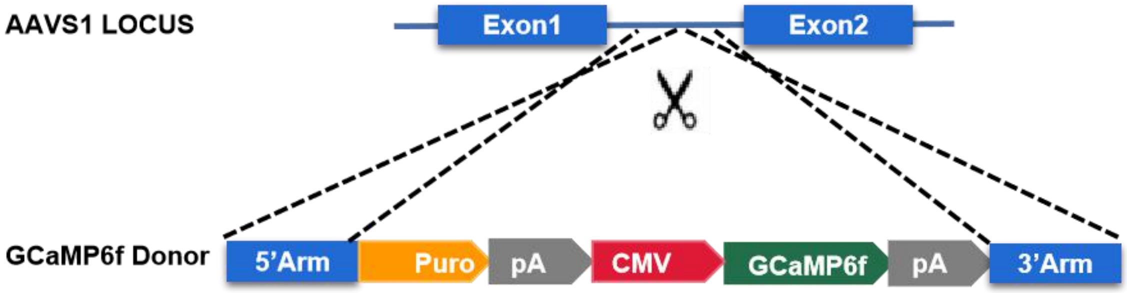

E:Space-averaged calcium transients showing parameters measured for analysis of calcium handling

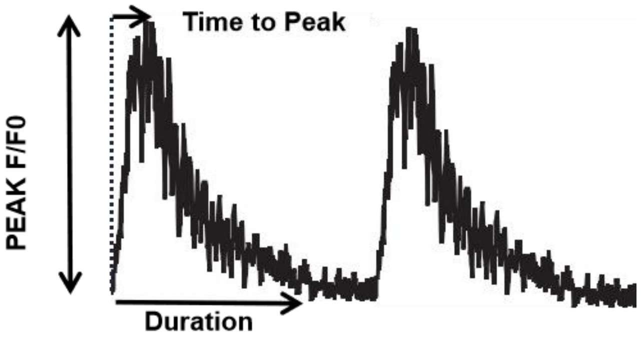

F:Sequence chromatograms demonstrate a Heterozygote *PLN* gene knockout line which one allely deleted 7 nucleotide(ACTTGCT).

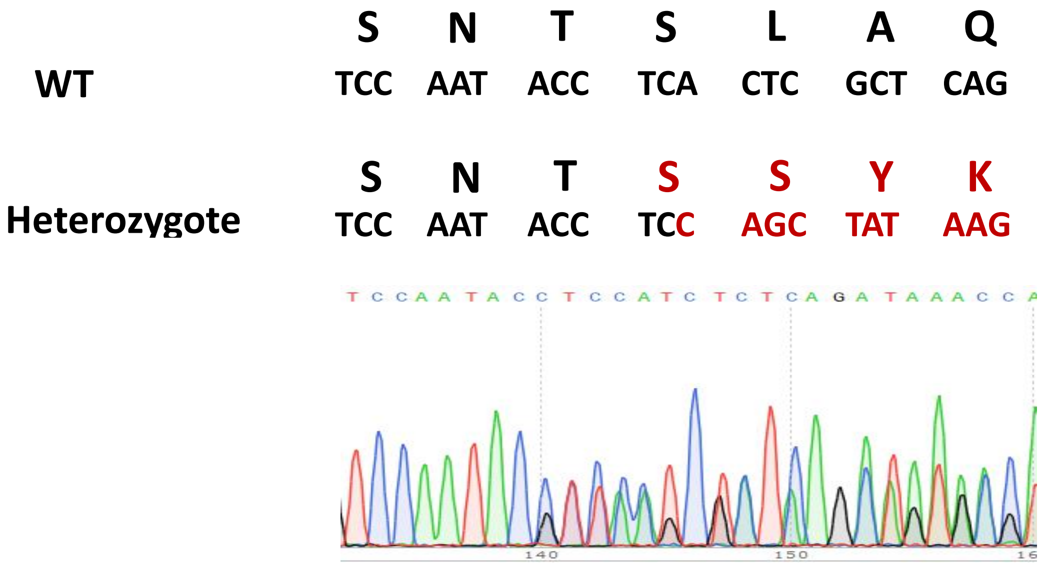

G:Quantification of *PLN* normalized by GAPDH in WT and *PLN* heterozygote hiPSC-CMs

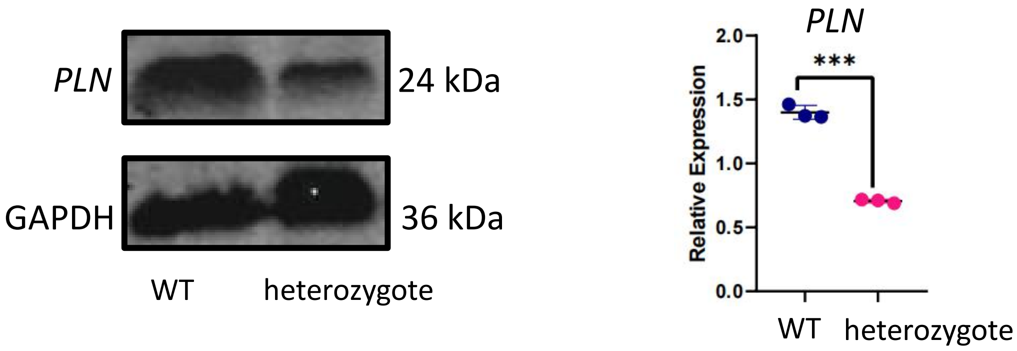

Supplementary Figure2:（A-D）the results of contractility in the presence of WT , *PLN*-KO,WT+ISO(500nM) and *PLN*-KO+ISO(E-H)the results of calcium transient in the presence of WT,*PLN*-KO,WT+ISO(500nM) and *PLN*-KO+ISO

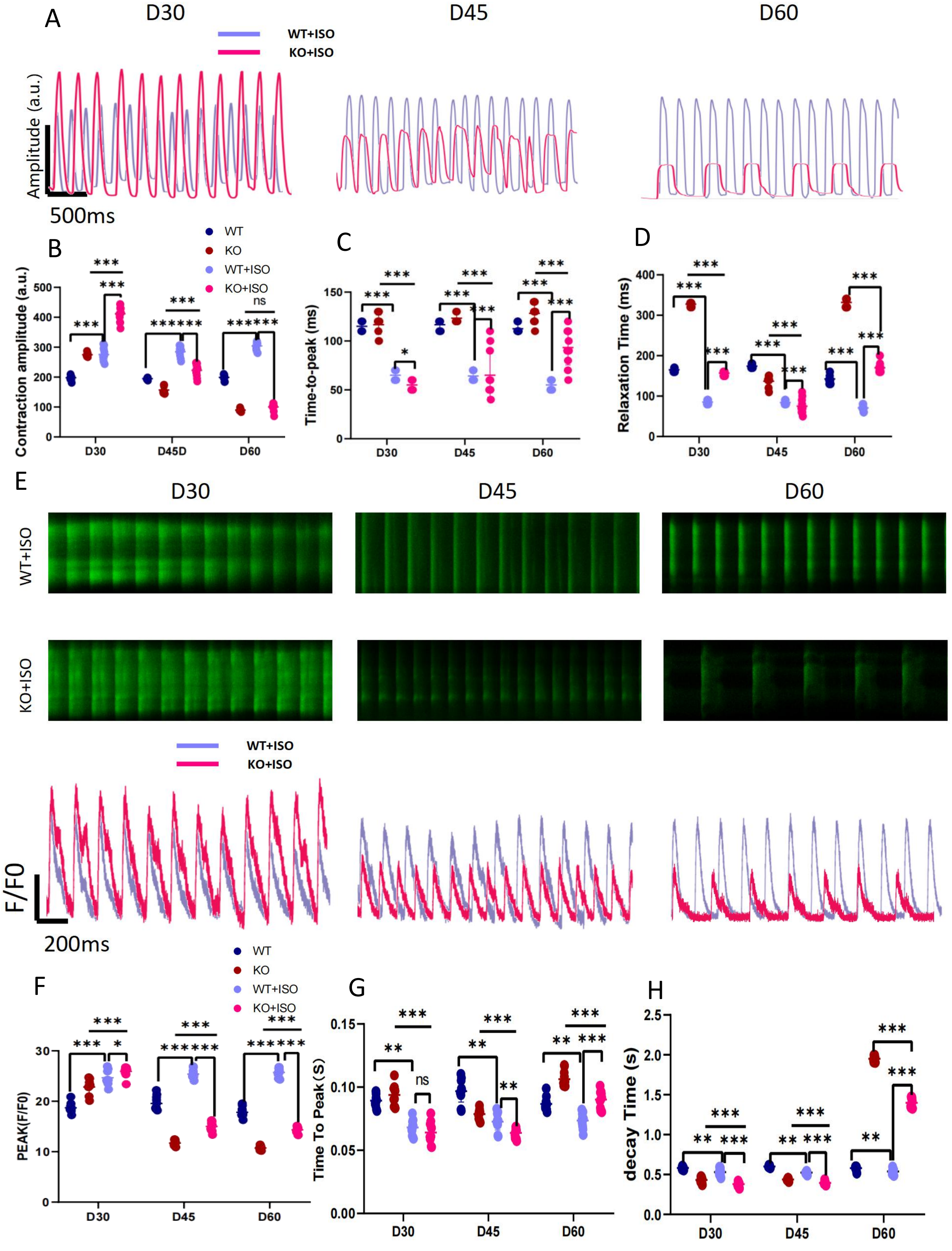

Supplementary Figure 3:(A) The Relative protein expression levels of cl-PARP /p-AKT/AKT/BCL-2/ cl-caspapse3 /bax at day 60;(B)The Relative protein expression levels of cl-PARP /p-AKT/AKT/BCL-2/ cl-caspapse3 /BAX with ranolazine at day 60（C）A Gene Set Enrichment Analysis (GSEA) revealed that pathways

A:

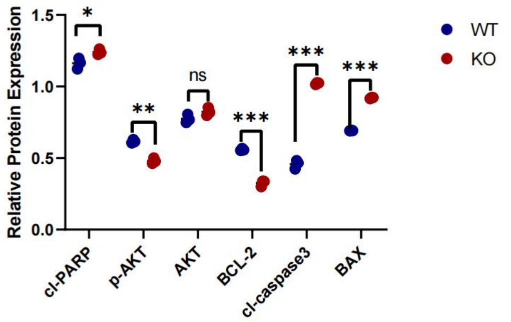

B:

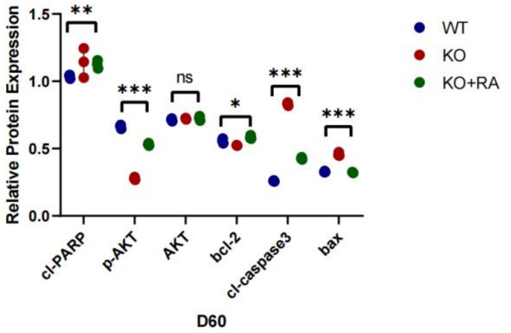

C:

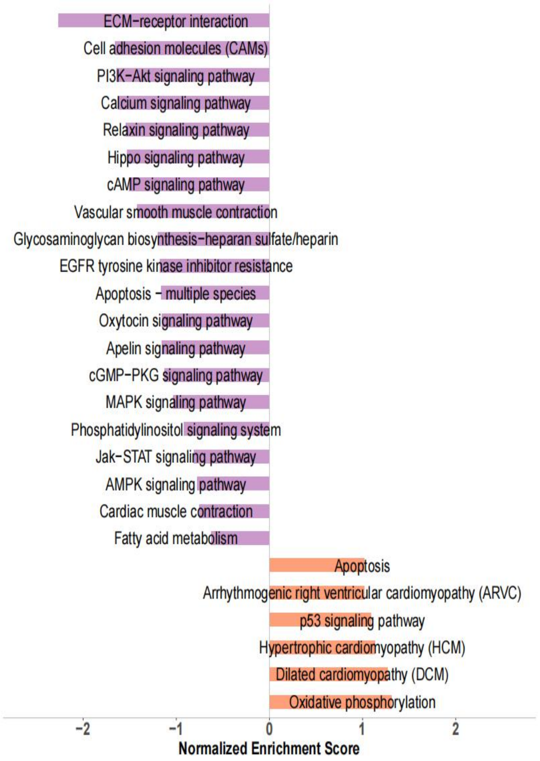

Supplementary Figure 4. Ranolazine recovered calcium transport disorders and myocardial contractility in *PLN*-KO hiPSC-CMs at day 30d and day 45.

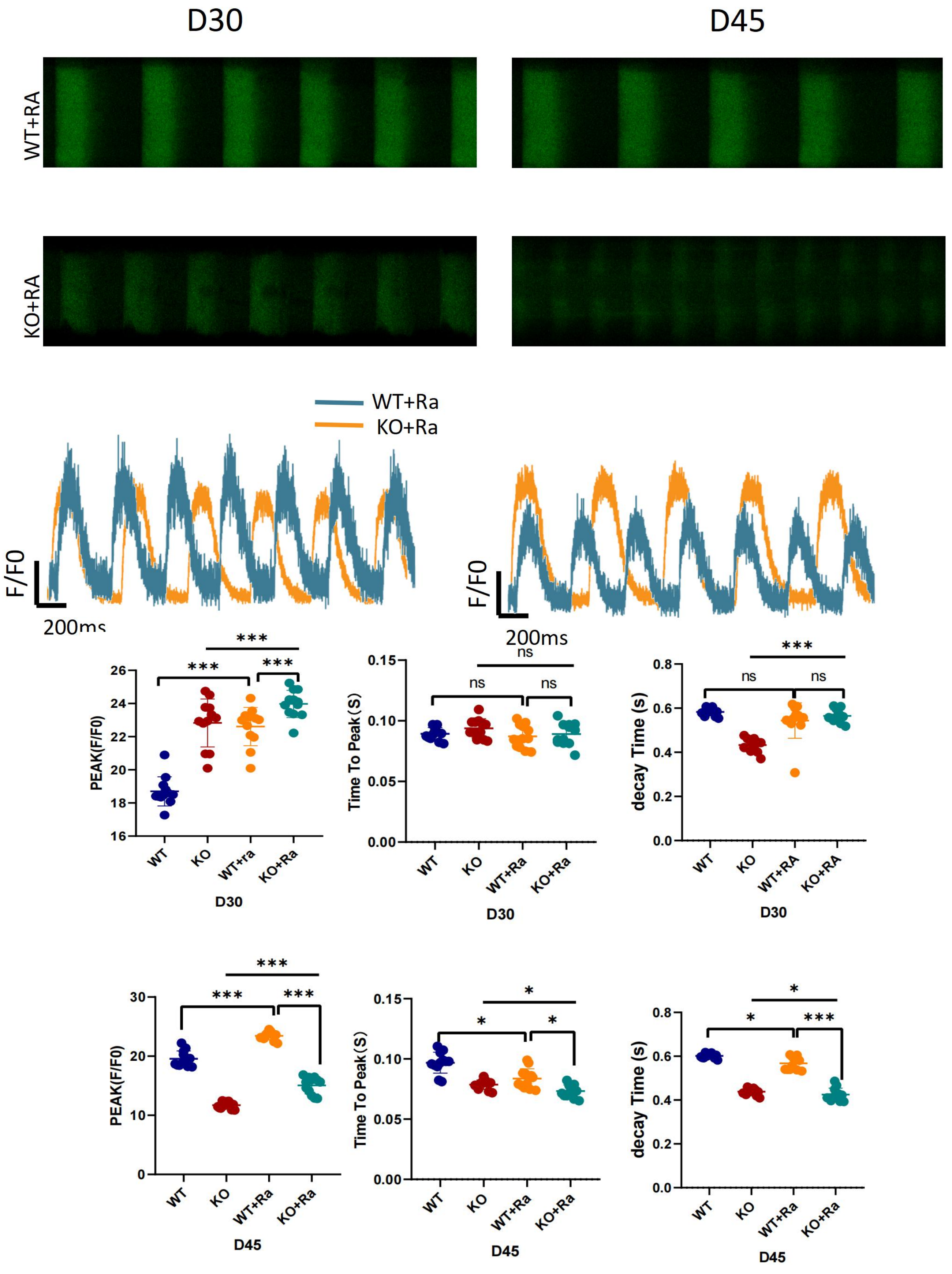

Supplementary Figure 5:Ranolazine recovered mitochondrial dysfunction and cell activates oxidative stress in *PLN*-KO hiPSC-CMs (a,b)Assessment of oxygen consumption rate (OCR) was performed with the application of compounds to assess ATP production (Oligomycin), maximum (Max) respiration (FCCP, carbonyl cyanide-4 [trifluoromethoxy] phenylhydrazone) and halted respiration (antimycin A and rotenone) for WT,*PLN* KO,WT+ranolazine and *PLN* KO+ranolazine hiPSC-CMs at day 30 and day 45(n=12)（C）Basal respiration of WT,*PLN* KO,WT+ranolazine and *PLN* KO+ranolazine hiPSC-CMs at day 30,45,60 (D) maximum respiration of WT,*PLN* KO,WT+ranolazine and *PLN* KO+ranolazine hiPSC-CMs at day 30,45,60(J) the ATP level in WT and *PLN* KO hiPSC-CMs at day 30,45,60.（E-J）The relative expression of glucose metabolism and fatty acid metabolism with ranolazine at day 60.

A:

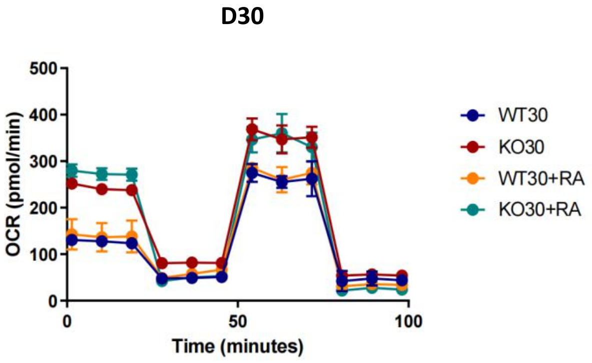

B:

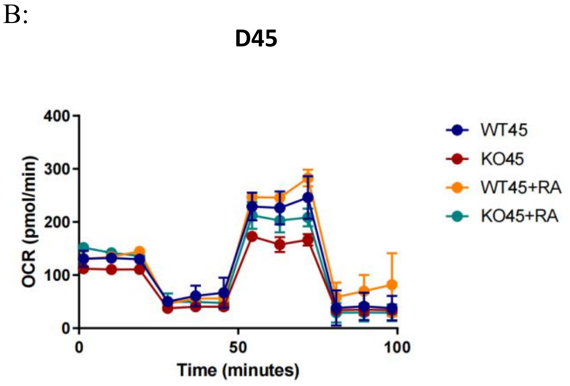

C

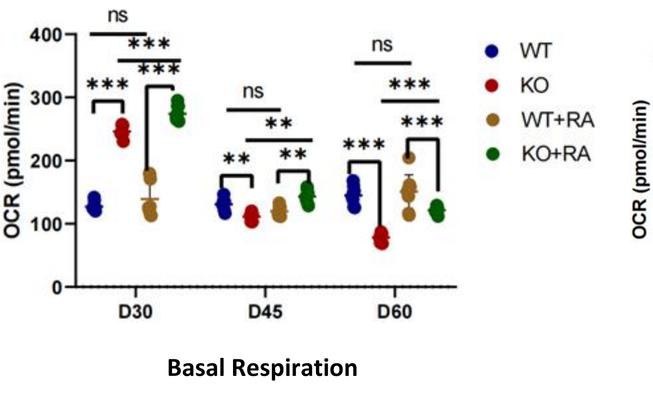

D:

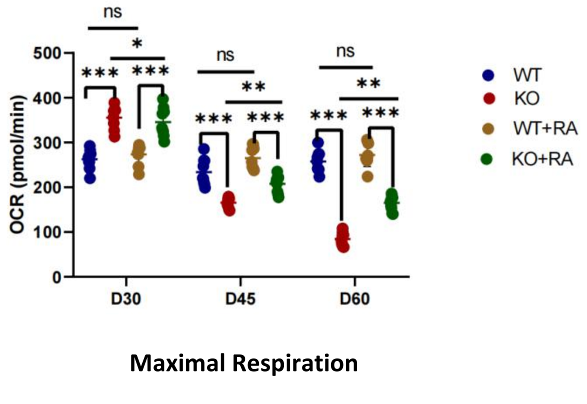

E

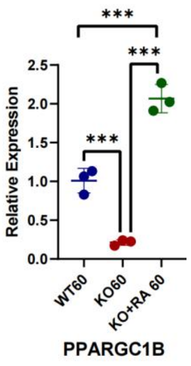

F

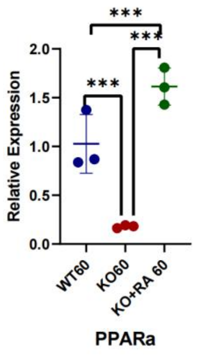

G

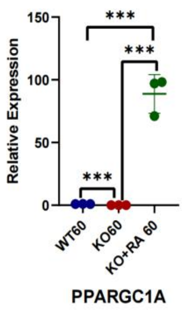

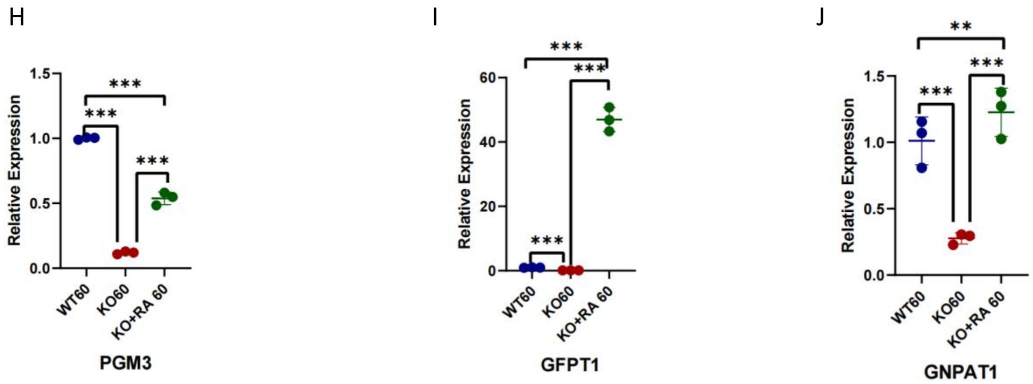
H
I
J
Relative Expression
1.5
1.0
0.5
0.0
60
40
20
0
***
**
WT60
KO60
KO+RA 60
PGM3
GFPT1
GNPAT1

Supplementary Figure 6:(A)Representative line-scan images from WT , *PLN* heterozygotes (+/-) hiPSC-CMs after 1 week of 1 μ M ISO treatment beginning at day 53 of cardiac differentiation. (B-D)Quantifification of peak, time to peak, and calcium decay time in WT-GCaMP+ISO and *PLN* heterozygotes（+/-）-GCaMP+ISO hiPSC-CMs at day 60 (n = 12 cells per group);（E）Representative line-scan images from WT+ISO , *PLN* heterozygotes (+/-) +ISO hiPSC-CMs after 5 day of 1 μ M ranolazine treatment.(F-H)Quantifification of peak, time to peak, and calcium decay time in WT,*PLN* heterozygotes+ISO,WT-GCaMP+ISO+Ra and *PLN* heterozygotes（+/-）-GCaMP+ISO+Ra hiPSC-CMs at day 60 (n = 12 cells per group);

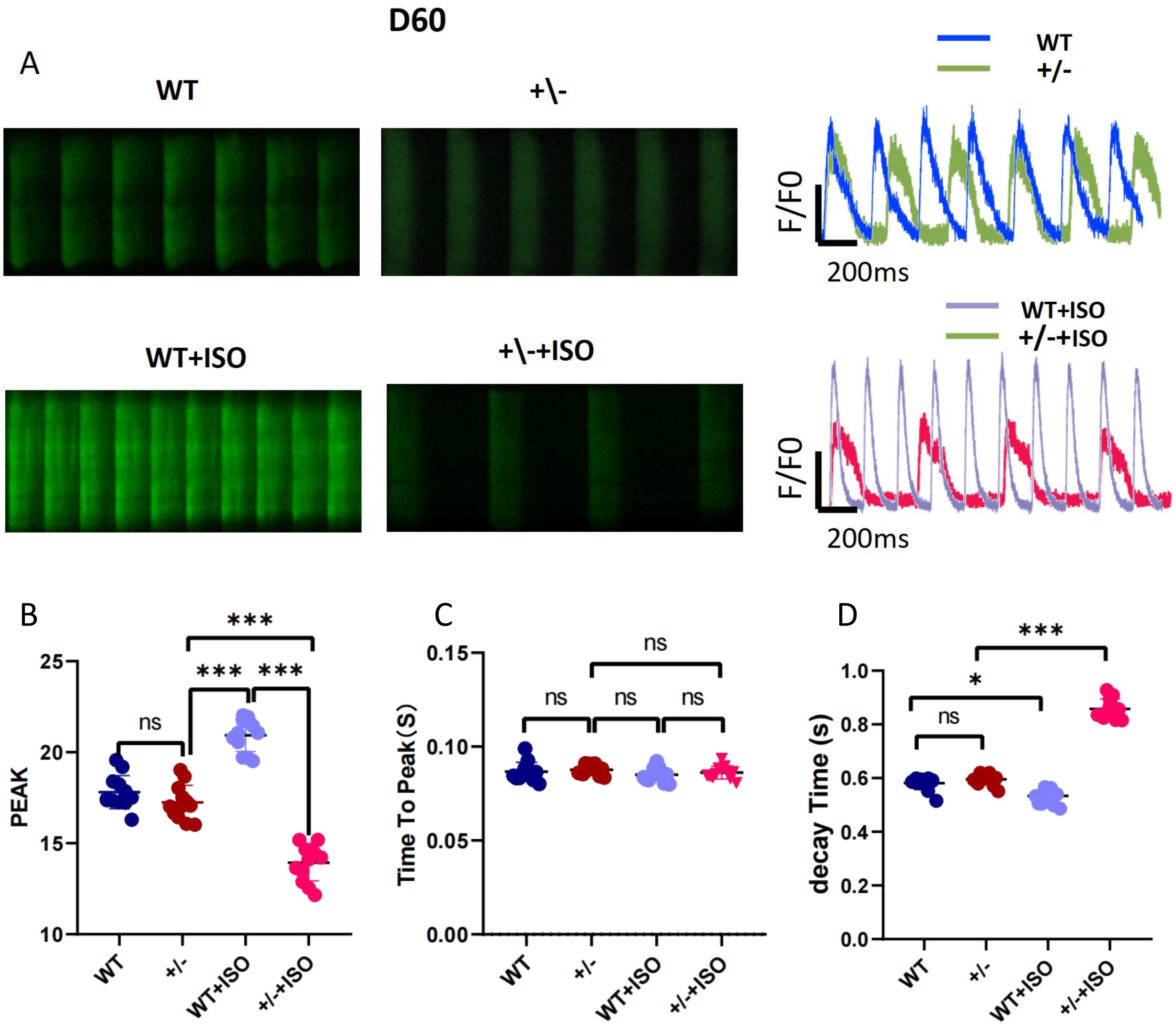

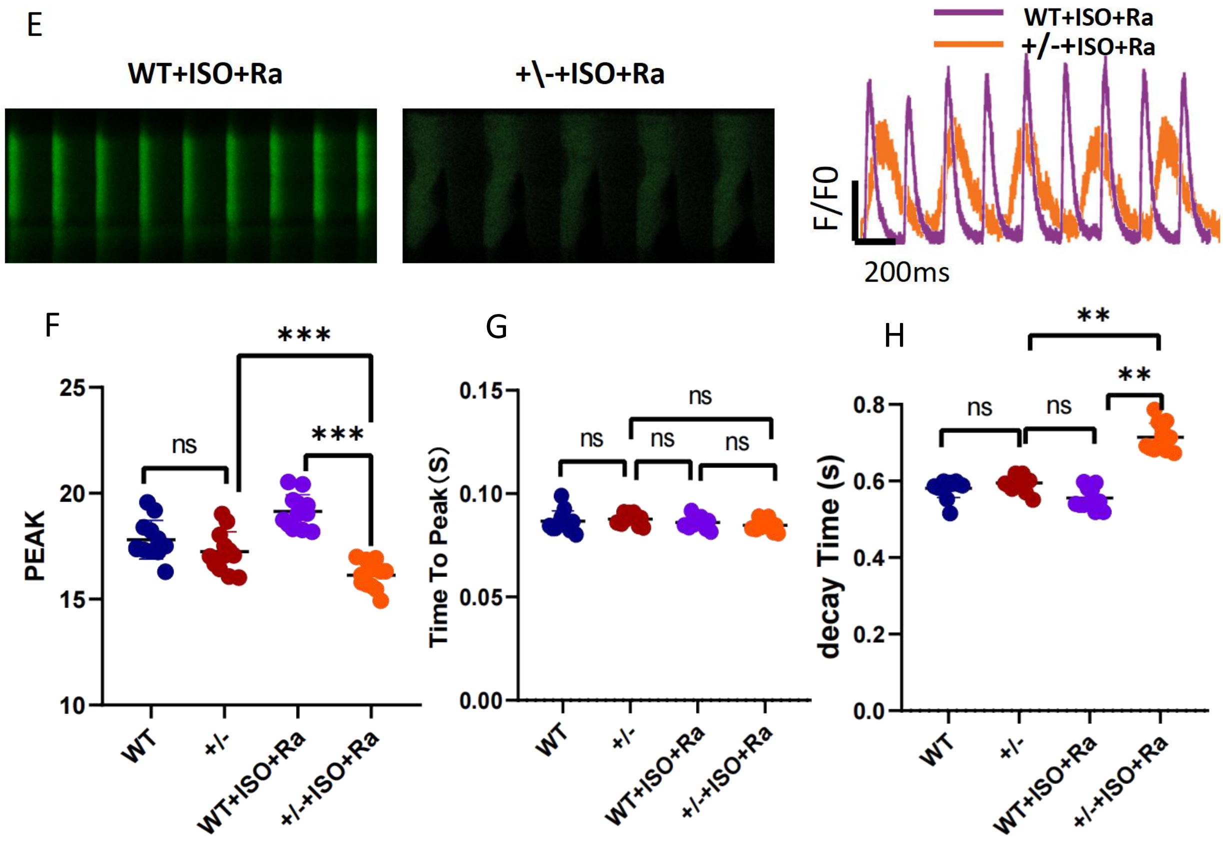

E
WT+ISO+Ra
+\-+ISO+Ra
WT+ISO+Ra
+/-+ISO+Ra
F/F0
200ms
F
PEAK
25
20
15
10
ns
***
***
WT
+/-
WT+ISO+Ra
+/-+ISO+Ra
G
Time To Peak (S)
0.15
0.10
0.05
0.00
ns
ns
ns
ns
WT
+/-
WT+ISO+Ra
+/-+ISO+Ra
H
decay Time (s)
0.8
0.6
0.4
0.2
0.0
ns
ns
**
**
WT
+/-
WT+ISO+Ra
+/-+ISO+Ra

Table1：Primer sequences used for q-PCR

Gene	Forward 5'-3'	Reverse 5'-3'
ND1	ATGGCCAACCTCCTACTCCTCATT	TTATGGCGTCAGCGAAGGGTTGTA
ND2	CCATCTTTGCAGGCACACTCATCA	ATTATGGATGCGGTTGCTTGCGTG
ACTB	CATGTACGTTGCTATCCAGGC	CTCCTTAATGTCACGCACGAT
MYH6	TCTCCGACAACGCCTATCAGTAC	GTCACCTATGGCTGCAATGCT
MYH7	GGCAAGACAGTGACCGTGAAG	CGTAGCGATCCTTGAGGTTGTA
ANP	ACAATGCCGTGTCCAACGCAGA	CTTCATTCGGCTCACTGAGCAC
BNP	TCTGGCTGCTTTGGGAGGAAGA	CCTTGTGGAATCAGAAGCAGGTG
TNNT2	TTCACCAAAGATCTGCTCCTCGCT	TTATTACTGGTGTGGAGTGGGTGTGG
ACTA1	AGGTCATCACCATCGGCAACGA	GCTGTTGTAGGTGGTCTCGTGA
RYR2	AGAACTTACACACGCGACCTG	CATCTCTAACCGGACCATACTGC
PLN	AGCACGTCAAAAGCTACAGAATCT	CTGATGTGGCAAGCTGCAGATC
GAPDH	*GGAGCGAGATCCCTCCAAAAT*	*GGCTGTTGTCATACTTCTCATGG*
PPARA	*TCGGCGAGGATAGTTCTGGAAG*	*GACCACAGGATAAGTCACCGAG*
PPARGC1A	*CCAAAGGATGCGCTCTCGTTCA*	*CGGTGTCTGTAGTGGCTTGACT*
PPARGC1B	*TGAGCAGACCTTGACAGTGGAG*	*GACTATGCTTGATGTCTGGTTTGA*
GNPAT1	*CCAACACATCCTGGAGAAGGCT*	*GGCTGACAACTCCAGTCTCTGT*
PGM3	*GGAGGCAATCAATGACCTGGTG*	*CCAAGCTCACTTCATGTGCAAGG*
GFPT1	*CCCTCTGTTGATTGGTGTACGG*	*GGAAAAGGCAGGTTGTGCTGTC*

Table2：Primary and Secondary Antibodies

Type	Antibody	Application	Dilution	Species	Manufacturer And Catalog Number
Primary	Anti-OCT4	Immunofluorescence	1:100	Rabbit Polyclonal	Santa Cruz sc-9081
	Anti-TRA-1-81	Immunofluorescence	1:100	Mouse Monoclonal	Santa Cruz sc-21706
	Anti-BAX	Western blot	1:1000	Rabbit Polyclonal	Abcam Ab182733
	Caspase3/P17/19 Polyclonal antibody	Western blot	1:1000	Rabbit Monoclonal	proteinTech 19677-1-AP
	PARP1 Polyclonal antibody	Western blot	1:1000	Rabbit Monoclonal	proteinTech 13371-1-AP
	Anti-*PLN*	Immunofluorescence	1:100	Mouse Monoclonal	Santa Cruz sc-393990
		Western blot	1:1000		
	Anti-cTnT	Immunofluorescence	1:100	Mouse Monoclonal	Abcam Ab8295
		Western blot	1:1000		
		Flow cytometry	1:200		
	AKT Polyclonal antibody	Western blot	1:1000	Rabbit Monoclonal	proteinTech 10176-2-AP
	Phospho-AKT(Ser473) Monoclonal antibody	Western blot	1:1000	Mouse Monoclonal	proteinTech 66444-1-Ig
	BCL-2 Polyclonal antibody	Western blot	1:1000	Rabbit Polyclonal	proteinTech 12789-1-AP
	Anti-GAPDH	Western blot	1:1000	Mouse Monoclonal	Santa Cruz sc-365062
Secondary	Goat anti-Mouse IgG Alexa Fluor 594	Immunofluorescence	1:200	Goat anti-Mouse IgG	Invitrogen A21145
	Goat anti-Rabbit IgG Alexa Fluor 488	Immunofluorescence	1:200	Goat anti-Rabbit IgG	Invitrogen A32731
	Chicken anti-Rabbit IgG	Immunofluorescence	1:200	Chicken anti-Rabbit IgG	Invitrogen A21442

Secondary	Alexa Fluor 594				
	Chicken anti-Mouse IgG Alexa Fluor 488	Immunofluorescence	1:200	Chicken anti-Mouse IgG	Invitrogen A21200
	Goat anti-Rabbit IgG (H + L) IRDye 800CW	Western blot	1:20000	Goat anti-Rabbit IgG	LI-COR 926-32211
	Goat anti-Mouse IgG (H + L) IRDye 800CW	Western blot	1:20000	Goat anti-Mouse IgG	LI-COR 926-32210

Brief Report

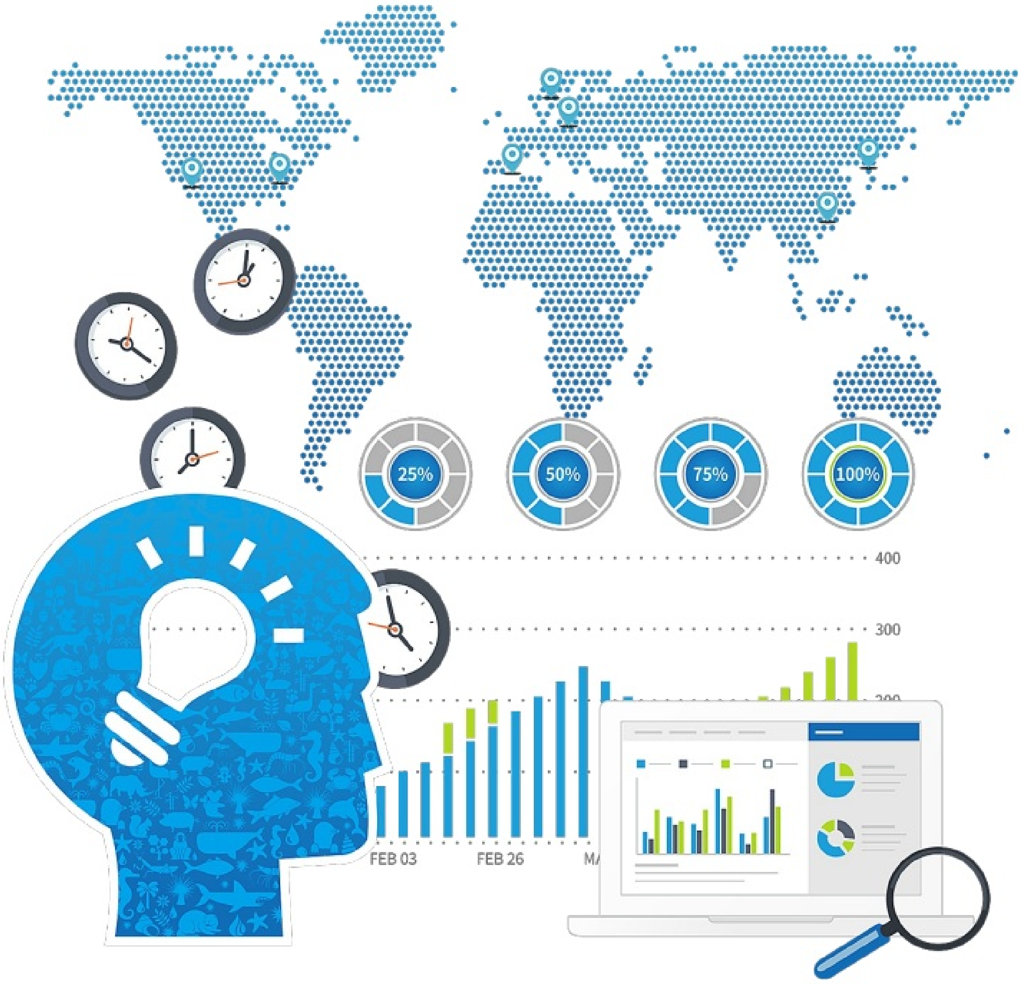

Catalogue

Catalogue 1
1 Project overview 2
1.1 Abstract 2
1.2 Analysis Plan 2
1.2.1 Setting of sample distribution 2
1.2.2 Differential grouping set 2
1.2.3 Time series analysis scheme 2
1.3 Sample correlation 3
1.3.1 Sample correlation 3
1.4 Expression quantity distribution 3
1.4.1 Boxplot 3
1.4.2 Gene expression density map 4
2 Reference Information 4
2.1 Reference species 4
2.2 RNA classification 4
2.3 Gene length 4
2.4 Number of exons 5
3 Reads filtering 6
3.1 Quality statistics of filtered reads 6
3.1.1 Reads filtering 6
3.2 Statistics of raw data 7
4 Reads Align 7
4.1 Align to the reference genome 7
4.2 Align to the reference gene 8
5 Significant DEGs 9

1 Project overview

Project number: F20FTSCCWLJ6851_HOwksE

1.1 Abstract

In this project, we sequence 12 samples used BGISEQ platform, averagely generating about 6.66G Gb bases per sample. The average mapping ratio with reference genome is 87.77%, the average mapping ratio with gene is 68.68%; 18191 genes were identified.

1.2 Analysis Plan

1.2.1 Setting of sample distribution

Biological experiments generally have biological replicates. Biological replicates generally occurs in biological experiment, and the multiple samples of biological replicates shall be classified into one group. The following table is the grouping of samples used in analysis:

ID	Group Name	Sample Name
1	WT45D	WT_45D_2,WT_45D_1,WT_45D_3
2	KO60D	KO_60D_1,KO_60D_2,KO_60D_3
3	WT60D	WT_60D_2,WT_60D_1,WT_60D_3
4	KO45D	KO_45D_2,KO_45D_1,KO_45D_3

1.2.2 Differential grouping set

One of the important analytic points of transcriptome sequencing is the comparison of the differences in gene expression. The control and treatment settings in pairwise comparison are as follows:

ID	Control Group	Treat Group
1	WT45D	WT60D
2	WT60D	KO60D
3	WT45D	KO45D
4	KO45D	KO60D

1.2.3 Time series analysis scheme

The correspondence between the name of the time series analysis plan and the sample:

No Result

1.3 Sample correlation

In order to reflect the correlation of gene expression between samples, the Pearson correlation coefficients of all gene expressions between each two samples were calculated, and these coefficients were reflected in the form of a heatmap. The correlation coefficients can reflect the similar situation of the overall gene expression between each sample. The higher the correlation coefficient is, the more similar the gene expression level is.

1.3.1 Sample correlation

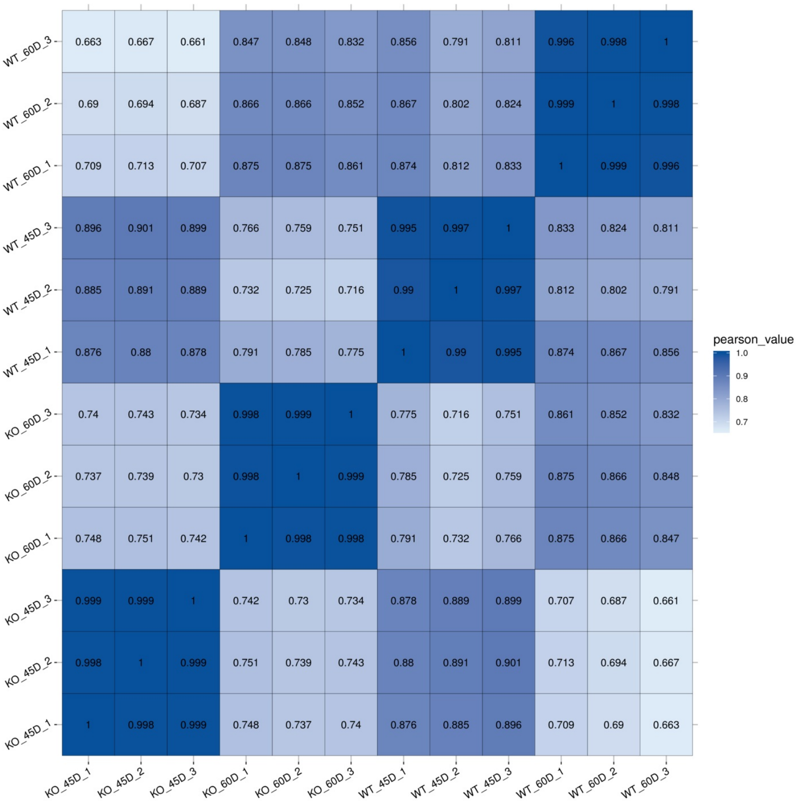

1.4 Expression quantity distribution

1.4.1 Boxplot

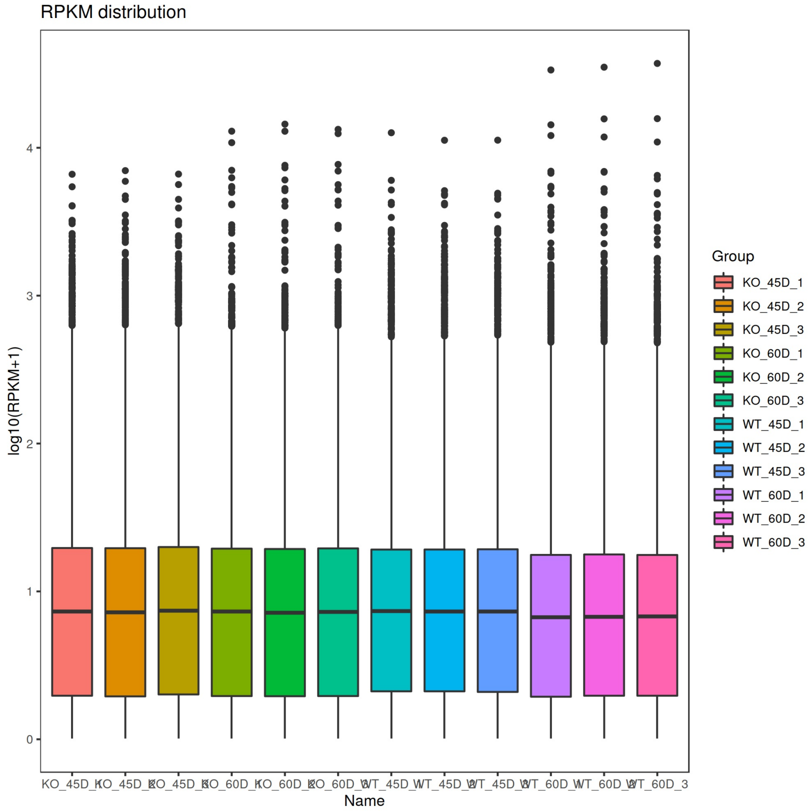

1.4.2 Gene expression density map

To show the gene amount under different FPKM value, we calculate the gene amount under three different FPKM ranges(FPKM <= 1、FPKM 1~10、FPKM >= 10).

No Result

2 Reference Information

2.1 Reference species

Species: Homo_sapiens

Source: NCBI

Reference Genome Version: GCF_000001405.38_GRCh38.p12

2.2 RNA classification

mRNA Num	lncRNA Num	miRNA Num
230487	177880	2656

2.3 Gene length

RNA Length	All	mRNA	mRNA	lncRNA	lncRNA

Item	RNA	Known	Novel	Known	Novel
0-500	53781	461	3846	34654	14820
500-1000	67098	5284	8233	42293	11288
1000-1500	40333	9629	8374	16902	5428
1500-2000	35301	13624	7816	11384	2477
2000-2500	31335	14418	7122	8484	1311
2500-3000	26160	13114	6628	5615	803
3000-3500	21427	10949	5945	4042	491
3500-4000	18783	9958	5443	3071	311
4000-4500	15620	8298	4805	2282	235
4500-5000	12960	6596	4440	1749	175
5000-5500	10917	5433	3933	1428	123
5500-6000	9237	4539	3551	1056	91
6000-6500	8133	4008	3123	921	81
6500-7000	6692	3063	2770	783	76
7000-7500	5673	2476	2516	616	65
7500-8000	5039	2260	2218	523	38
8000-8500	4427	1870	2030	483	44
8500+	35451	7744	23970	3457	280

2.4 Number of exons

Exon Num Item	All RNA	mRNA Known	mRNA Novel	lncRNA Known	lncRNA Novel
1	61395	1513	7643	25720	26519
2	60469	3231	10986	39494	6758
3	46715	5136	7913	31409	2257
4	32360	6819	7042	17448	1051

5	24276	7467	6537	9679	593
6	19218	7529	5933	5434	322
7	15897	7211	5397	3086	203
8	13975	7014	4955	1877	129
9	12642	6785	4491	1273	93
10+	121420	71019	45866	4323	212

3 Reads filtering

Sequencing Platform: BGISEQ

Sequencing length: PE150

The raw data for sequencing contains reads of low quality, connector contamination, and excessively high levels of unknown base N. These reads need to be removed before data analysis to ensure the reliability of the results.

3.1 Quality statistics of filtered reads

3.1.1 Reads filtering

Sample Name	Total Raw Reads	Total Clean Reads	Total Clean Bases	Clean Reads Q20	Clean Reads Q30	Clean Reads Ratio
KO_45D_1	45.57	44.61	6.69	94.63	88.17	97.88
KO_45D_2	45.57	44.47	6.67	94.44	87.96	97.57
KO_45D_3	44.13	42.53	6.38	94.53	88.12	96.37
KO_60D_1	45.57	44.53	6.68	94.42	87.89	97.72
KO_60D_2	49.08	44.62	6.69	94.90	88.93	90.91
KO_60D_3	47.33	45.43	6.81	94.63	88.38	95.99
WT_45D_1	45.57	44.66	6.70	94.62	88.13	97.99
WT_45D_2	45.57	43.90	6.58	95.03	88.98	96.32
WT_45D_3	47.33	45.42	6.81	94.70	88.51	95.96
WT_60D_1	45.57	44.48	6.67	94.52	88.07	97.61

WT_60D_2	45.57	44.33	6.65	94.68	88.41	97.28
WT_60D_3	45.57	44.16	6.62	94.45	87.97	96.91

3.2 Statistics of raw data

Sample Name	N Read Num	Adapter Read Num	Low Quality Read Num	Clean Read Num
WT_45D_2	161510	1513732	102	43898548
KO_45D_3	197292	1403626	136	42530452
WT_60D_2	198608	1041796	116	44333372
WT_45D_3	204096	1705868	104	45416666
WT_45D_1	149598	767990	128	44656176
KO_60D_1	193790	847398	116	44532588
WT_60D_1	198856	891500	130	44483406
KO_45D_1	157244	809532	100	44607016
KO_60D_3	209530	1686102	120	45430982
KO_60D_2	209488	4251802	100	44618186
KO_45D_2	198394	909118	92	44466288
WT_60D_3	192948	1216288	106	44164550

4 Reads Align

4.1 Align to the reference genome

After getting clean reads, we used HISAT to align the clean reads to the reference genome. The alignment results are shown as follows.

Sample Name	Total Clean Read	Total Mapping Genome Ratio	Uniquely Mapping Genome Ratio
KO_45D_1	44.61	89.36	84.77
KO_45D_2	44.47	87.93	83.58

KO_45D_3	42.53	87.90	83.58
KO_60D_1	44.53	87.60	82.94
KO_60D_2	44.62	86.05	81.03
KO_60D_3	45.43	86.77	82.01
WT_45D_1	44.66	88.86	84.33
WT_45D_2	43.90	89.09	84.44
WT_45D_3	45.42	87.32	82.83
WT_60D_1	44.48	87.72	83.40
WT_60D_2	44.33	87.63	83.18
WT_60D_3	44.16	87.02	82.63

4.2 Align to the reference gene

Align clean reads to the reference genes by using Bowtie2 to get the alignment result.

Sample Name	Total Clean Read	Total Mapping Gene Ratio	Uniquely Mapping Gene Ratio
KO_45D_1	44.61	74.74	71.34
KO_45D_2	44.47	75.34	71.85
KO_45D_3	42.53	74.56	71.20
KO_60D_1	44.53	68.33	64.18
KO_60D_2	44.62	61.96	57.83
KO_60D_3	45.43	65.31	61.12
WT_45D_1	44.66	71.54	67.60
WT_45D_2	43.90	71.55	67.62
WT_45D_3	45.42	71.12	67.22
WT_60D_1	44.48	64.35	59.65
WT_60D_2	44.33	63.68	58.88
WT_60D_3	44.16	61.64	57.01

5 Significant DEGs

The statistical results of significant DEGS are as follows.

Diff Plan	Diff Num
WT60D-vs-KO60D	8475
WT45D-vs-WT60D	10168
WT45D-vs-KO45D	10712
KO45D-vs-KO60D	10476
